# Critical review on the stability and thermal conductivity of water-based hybrid nanofluids for heat transfer applications

**DOI:** 10.1039/d5ra00844a

**Published:** 2025-05-01

**Authors:** Mageswari Manimaran, Mohd Nurazzi Norizan, Mohamad Haafiz Mohamad Kassim, Mohd Ridhwan Adam, Norli Abdullah, Mohd Nor Faiz Norrrahim

**Affiliations:** a Bioresource Technology Division, School of Industrial Technology, Universiti Sains Malaysia Penang 11800 Malaysia mohd.nurazzi@usm.my; b Green Biopolymer, Coatings & Packaging Cluster, School of Industrial Technology, Universiti Sains Malaysia Penang 11800 Malaysia; c School of Chemical Sciences, Universiti Sains Malaysia Penang 11800 Malaysia; d Centre for Defence Foundation Studies, Universiti Pertahanan Nasional Malaysia Kem Perdana Sungai Besi 57000 Kuala Lumpur Malaysia; e Defence Research Institute, Universiti Pertahanan Nasional Malaysia Kem Perdana Sungai Besi Kuala Lumpur 57000 Malaysia faiz@upnm.edu.my

## Abstract

Thermal conductivity is undoubtedly the most significant physicochemical property for evaluating the thermal efficiency of nanofluids. In addition to thermal conductivity, stability is another crucial factor that must be assessed, as maintaining long-term stability in closed-circuit and continuous-cycle applications remains a major challenge. Notably, stability is closely linked to thermal conductivity in nanofluid applications. Recent studies on nanofluids have explored the incorporation of multiple types of nanoparticles known as hybrid nanofluids into heat transfer fluids such as water, ethylene glycol (EG), oil, and refrigerants. These hybrid nanofluids aim to enhance the thermal properties of conventional base fluids. However, nanoparticles at the nanoscale exhibit a strong tendency to aggregate owing to substantial van der Waals interactions, resulting in sedimentation and clogging, which compromise stability. This aggregation negatively impacts both stability and thermal conductivity. This review provides an in-depth analysis of the latest advancements in water-based hybrid nanofluids, incorporating various nanoparticle hybridizations, including metals, metal oxides, carbon-based materials, and plant-based nanomaterials such as nanocellulose in combination with synthetic nanoparticles, an area that remains relatively unexplored. Furthermore, this review discusses characterization techniques and strategies for improving the stability and thermal conductivity of hybrid nanofluids, including chemical modifications such as the addition of surfactants or surface functionalization as well as physical modifications such as optimizing the volume fraction of hybrid nanocomposites, selecting appropriate nanoparticle types and sizes, adjusting ultrasonication time, and modifying pH levels. This review is based on recent studies published between 2019 and 2024.

## Abstract 2

Thermal conductivity is undoubtedly the most significant physicochemical property for evaluating the thermal efficiency of nanofluids.

## Graphical abstract

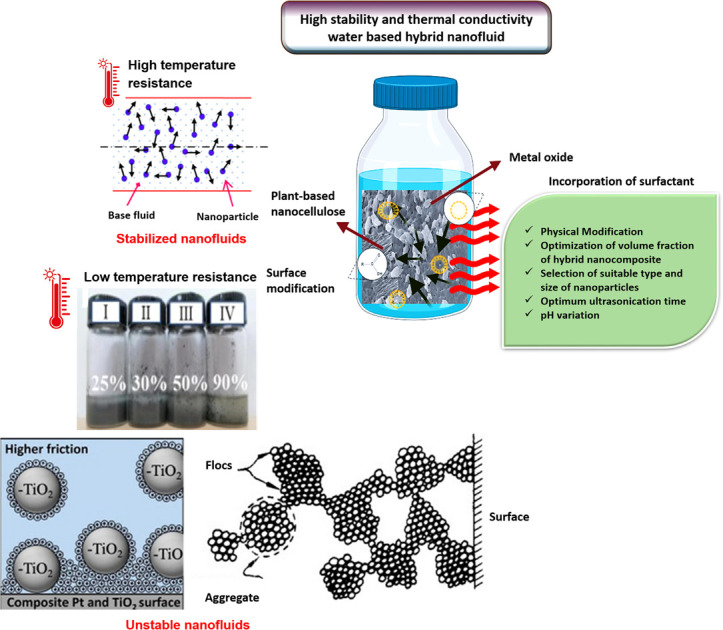

## 1 Introduction

The rapid increase in heat generation across several industries, including manufacturing, microelectronics, transportation, and thermal power plants, necessitates the use of efficient coolants or heat transfer fluids such as nanofluids for effective heat dissipation.^1^ Water is the most common, inexpensive and easily available heat transfer fluid and has been previously utilised in automobiles and heat exchangers owing to its low viscosity and high thermal capacity. Among other conventional heat transfer fluids, water has the highest thermal conductivity (0.598 W mK^−1^) at 20 °C, as shown in Table 1. Hybrid nanofluids with water as the base fluid are known for their high thermal conductivity and efficient heat dissipation, thereby enhancing overall system performance.

**Table 1:** Thermal conductivity of different types of base fluids

Type of base fluid	Thermal conductivity (W mK^−1^)
Water	0.598
Ethylene glycol	0.251
Engine oil	0.145
Propylene glycol	0.340
Refrigerants	0.1–0.2

In addition, water has the highest dielectric constant, indicating high repulsive potential. Since the repulsive potential and the base fluid's dielectric constant are closely correlated, more stability is predicted with higher dielectric constants. However, water has a high boiling point, low thermal conductivity, and can cause corrosion. As an alternative, nanofluid, which is a liquid suspension composed of water (base fluid) and nanoparticles with diameters smaller than 100 nm with or without surfactants, was invented to improve the thermal conductivity of the base fluid. The term “nanofluid” was first coined by Choi, in 1995, who created a unique liquid by adding copper (Cu) nanoparticles to water to increase the thermophysical properties of water.^2^

Various nanoparticles have been used to produce water-based nanofluids, including metal-based nanoparticles such as silver (Ag), Cu, and nickel (Ni) as well as metal oxide nanoparticles such as aluminium oxide (Al_2_O_3_), copper oxide (CuO), iron(iii) oxide (Fe_2_O_3_), magnesium oxide (MgO), silicon dioxide (SiO_2_), titanium dioxide (TiO_2_), and zinc oxide (ZnO) or their combination with carbon-derived nanoparticles such as carbon nanotubes (CNTs), multiwalled carbon nanotubes (MWCNTs), and graphene oxide (GO) owing to their high thermal conductivity and uniform distribution of nanoparticles in the base fluid, which can result in good heat transfer performance in many applications, including heat transfer systems such as in automobile radiators, refrigerators, heat pipes, and solar application.^3,4^ The benefit of using nano-sized (1–100 nm) particles in base fluids is that it can reduce the size of heat transfer systems and the energy required for fluid pumping, hence facilitating energy and cost savings in comparison to the use of micro-sized particles in traditional heat transfer fluids.^5^ Furthermore, nanoparticles with a large surface area can mitigate density disparities, allowing for stable suspension and serving as a substitute for microparticles, which have drawbacks such as channel blockage, abrasion, and pressure drop.^6–8^

**Fig. 1:**
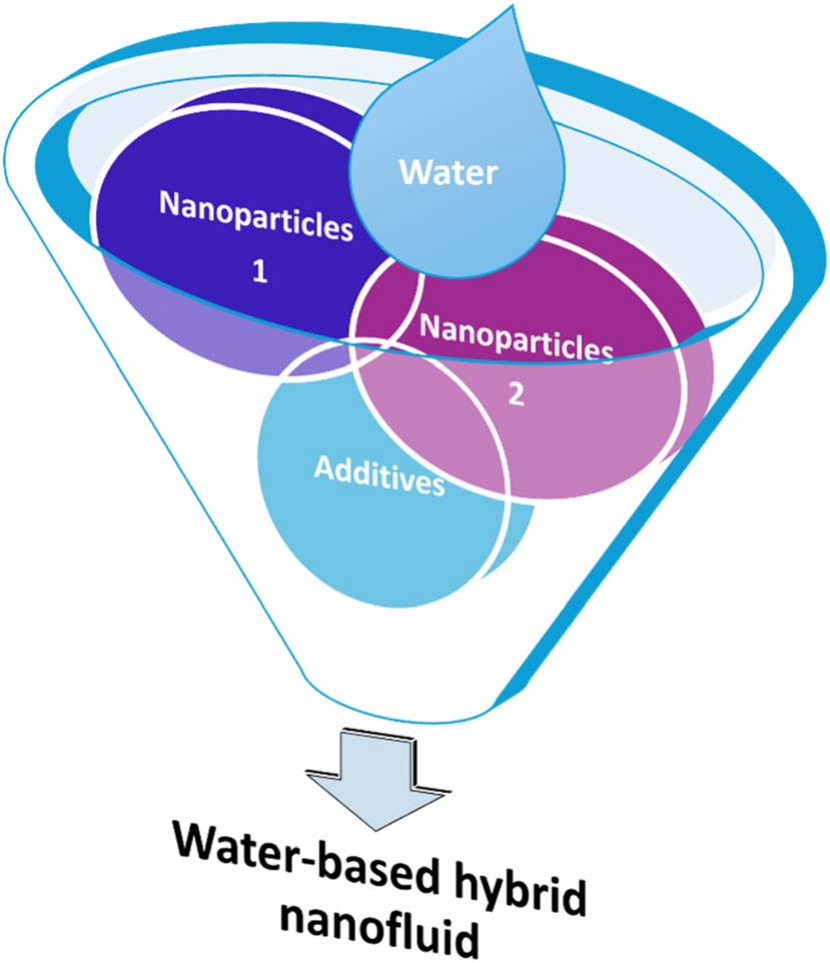
Basic components of water-based hybrid nanofluids.

However, recent research in nanofluids has explored the possibility of combining or mixing two or more different types of nanoparticles in base fluids such as water to create “hybrid nanofluids” to increase the thermal conductivity and stability of hybrid nanofluids,^9,10^ with some modification, as shown in Fig. 1. Apart from those nanoparticles mentioned before, plant-based nanomaterials called nanocellulose have also become popular nanoparticles lately that are used in hybrid nanofluids.^11^ Moreover, Sivasankaran and Bhuvaneswari^12^ have mentioned that hybrid nanofluids, which combine two or more types of nanoparticles, can improve the heat transfer coefficient by 4 to 6% compared to mono nanofluids. Additionally, Alklaibi *et al.*^13^ noted that hybrid nanofluids combining Fe_3_O_4_ and SiO_2_ offer higher thermal conductivity compared to mono nanofluids and base fluids. Nanoparticles within nanofluids boost thermal conductivity to the highest heat transmission level. Key factors in selecting the type of nanoparticles include cost, thermal stability, and transportation performance. As the next generation of heat transfer fluids, these innovative hybrid nanofluids show a lot of potential for use in heat transfer applications.

However, a significant issue with these types of nanoparticles is their tendency to aggregate, limiting dispersion and causing sedimentation, which renders them impractical for use in nanofluids.^14,15^ This is due to the high surface energy of the nanoparticles achieving stable hybrid nanofluids, which is a critical prerequisite before characterizing their thermophysical properties, necessitating the optimization of numerous parameters to attain stability.^16^ Large particles with non-uniform size distributions are particularly prone to aggregation due to attractive van der Waals forces, leading to the formation of clusters that hinder their application in heat transfer processes and pose practical challenges.

Therefore, ensuring stable nanofluids requires understanding and optimizing the major variables that influence the total interaction forces, including double-layer repulsion and van der Waals attraction, between suspended nanoparticles.^17^ According to the Derjaguin–Landau–Verwey–Overbeek (DLVO) theory, the low stability of hybrid nanofluids results from particle agglomeration driven by the dominant van der Waals attractive potential over the electrostatic repulsive potential. Stability is paramount for nanofluids in heat transfer applications, as it profoundly impacts their thermal conductivity.^18^ Long-term stability combined with high thermal conductivity is crucial for the commercial viability of nanofluids.^19^ Aggregation and sedimentation are critical factors influencing the stability and physicochemical properties of nanofluids.^20^ The stability of hybrid nanofluids hinges on interactions between nanoparticles and the base fluid, influenced by chemical and physical factors such as pH, surfactants, and production methods.^16^ The reduced thermal efficiency observed in some nanofluids during heat transfer applications often correlates with poor dispersion stability.

There is a notable lack of comprehensive studies examining the interplay between stability and thermal conductivity in hybrid nanofluids. To address this gap, this study aims to provide a thorough review of the existing literature, focusing specifically on stability and thermal conductivity with the combination of different types of nanoparticles including the bio-based nanoparticles called nanocellulose in water-based hybrid nanofluids and the relationship between the stability and thermal conductivity based on specific testing or characterization such as zeta potential, particle distribution, sediment observation and also KD2 Probe for stability and thermal conductivity evaluation, respectively. This review encompasses only experimental investigations, emphasizing stability and thermal conductivity, within the broader context of water-based hybrid nanofluid properties.^21^ Additionally, it examines strategies employed to enhance the stability and thermal conductivity of water-based hybrid nanofluids, offering insights into current research and future directions in this field.

## 2 Water as a heat transfer fluid

**Fig. 2:**
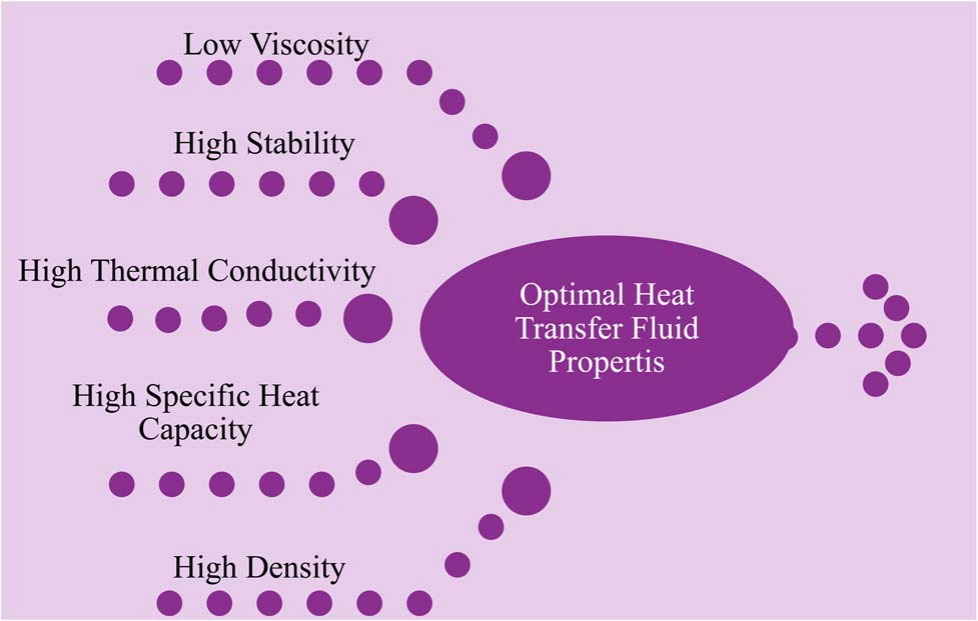
Optimal properties of a heat transfer fluid.

The selection of a base fluid is very important as it affects thermal stability, heat transfer efficiency, and expansion rates. The optimal heat transfer fluid should possess properties such as high stability, density, thermal conductivity, specific heat, and low viscosity,^22,23^ as shown in Fig. 2. A heat transfer fluid with an adequate combination of these properties can improve heat transfer performance over a long period of operating temperatures and flow rates. Achieving a highly stable heat transfer fluid is crucial, as it ensures efficient heat transfer over an extended period and reduces the risk of system damage or safety problems.^24^

In addition, a base fluid with low viscosity increases the thermal conductivity of nanofluids by allowing particles to interact more freely, resulting in better Brownian motion of the nanoparticles and increased interaction over time. Water has the lowest dynamic viscosity of any basic fluid, measuring 1.0 mPa s at 20 °C and dropping to 0.3543 mPa s at 80 °C. Furthermore, several studies have been undertaken to find base fluids that perform exceptionally well with specific nanoparticles.^25^ The best option is a water-based fluid, as it is the most efficient, easy to handle in the lab or on an industrial scale, and neutral to most types of nanoparticles at temperatures below 100 °C. Moreover, water is an excellent choice for heating and cooling due to its large heat capacity and strong thermal conductivity compared to other heat transfer fluids. However, water quality and source should be taken into account when using it for nanofluid applications. For instance, while tap water is affordable and practical, it likely contains contaminants that might block fluid channels or cause corrosion. To ensure the system functions as intended, it is crucial to employ high-quality water treatment. However, water freezes quickly and has a low boiling point, making it unstable and challenging to control under extreme conditions.^26^ Therefore, water is often mixed with glycol (normally 50/50, 60/40, or 70/30 glycol to water) to provide high stability at higher temperatures in heat transfer applications, as glycol can lower the freezing temperature, increase the boiling point, and reduce corrosion as an inhibitor. It is typically mixed with water to produce EG or propylene glycol (PG).

EG has a high boiling point and a low freezing point, making it more suitable for chillers designed to operate under low-temperature conditions. It also has better thermal stability over a wide temperature range, as well as high specific heat and thermal conductivity. In contrast, PG has a lower specific heat and a lower risk of toxicity, making it appropriate for use in food and beverage applications and in limited spaces. The combination of water and EG can be denoted as a base fluid with numerous attractive thermal properties such as high boiling point, low freezing point, stability over a wide temperature range, and low viscosity, which reduces the pump requirement.^27^ It is essential to use the lowest possible quantity of EG to meet the requirements, as the performance of the heat transfer fluid decreases with the increase in EG content. The thermal conductivity of nanofluids increases when the water-to-EG volume ratio increases.

## 3 Hybrid nanofluid preparation

**Fig. 3:**
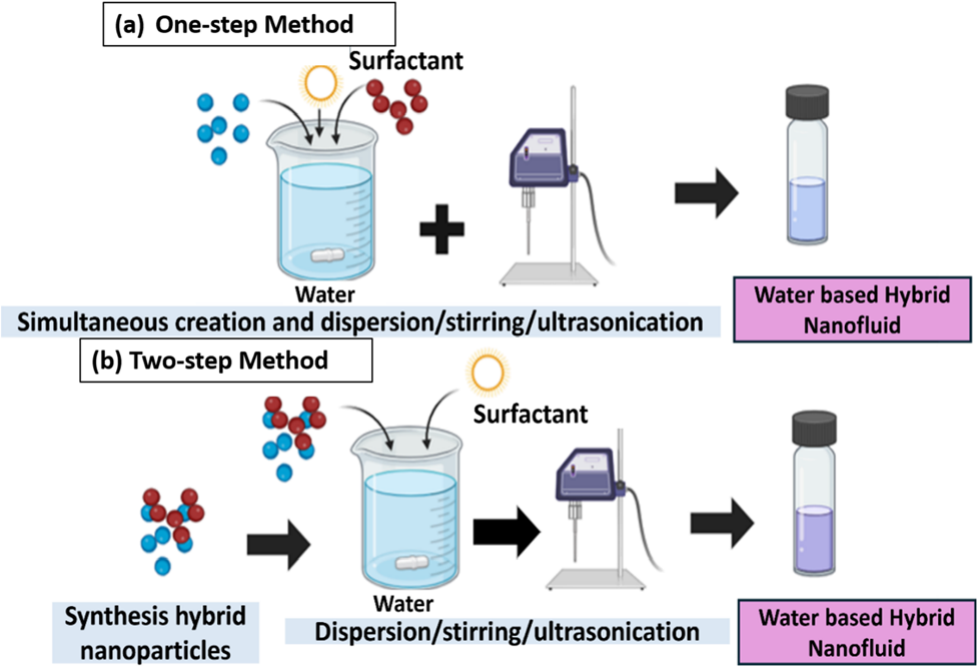
Differences between the (a) one-step and (b) two-step methods.

The addition of more than one type of nanoparticle into a single element, known as hybridization, results in superior properties and characteristics. In general, there are two ways to prepare a hybrid nanofluid: one-step and two-step methods.^28^ The two-step method is more suitable for oxide nanoparticle-based nanofluids, whereas the one-step method involves direct dispersion of metallic nanoparticles into a base fluid, as shown in Fig. 3(a). This method is considered one of the most effective for producing a consistent suspension of nanofluids with minimal particle agglomeration.^29^ However, the one-step approach can only be used to manufacture small amounts of nanofluids, which limits its application. Additionally, this method is highly risky because any contaminants can disrupt the system's operation, leading to decreased efficiency and reduced system life, such as in automobiles.

Comparatively, in the two-step method, the nanoparticles are produced or obtained separately and then suspended in the base fluid. To date, most studies have preferred the two-step method because it can be used for large-scale manufacturing.^30^ The first stage involves the synthesis hybrid nanocomposite with different combinations of nanoparticles. However, the nanocellulose-based nanocomposite can be prepared by *ex situ* preparation (nanoparticles separately synthesized and added to nanocellulose) or by *in situ* preparation (using nanocellulose as a template).^31^ In the second stage, these nanomaterials are dispersed in a heat transfer fluid using methods such as simple mixing, magnetic stirring, ultrasonication, high-shear mixing, homogenization, or a combination of these methods,^32^ as shown in Fig. 3(b), to stabilize the nanofluid. The duration of stirring directly affects the stability of nanoparticles in the fluid. The magnetic stirrer's force can break the weak bonds of agglomerated nanoparticles. This is necessary due to the high tendency of nanoparticles to agglomerate because of van der Waals attraction between particles.

However, the nanoparticles' large surface area and surface activity cause them to clump, making dispersion a crucial step. In some cases, surfactants are used to increase the stability of nanoparticles in the base fluid. Although the one-step process appears straightforward, the two-step procedure is better suited for large-scale nanofluid applications. Furthermore, this technology can be complemented by an effective continuous nanoparticle manufacturing method, which has been recently developed. When two or more distinct nanoparticles are combined to form hybrids, the resulting particles can have thermal conductivity comparable to that of single nanoparticles. The generation of stable hybrid nanofluids remains a challenge due to material selection and synthesis difficulties. The two-step preparation procedure is the most frequently utilized, according to the literature. For example, Suresh *et al.*^33^ used the two-step method to produce an Al_2_O_3_/Cu water-based hybrid nanofluid. However, the stability of the nanofluid relies on the production method. When a nanofluid is unstable, the Brownian motion of the particles is slowed, which hinders the fluid's ability to carry heat. Therefore, to reduce material costs and enable their use in commercial engineering, it is essential to create hybrid nanofluids using the appropriate approach.

## 4 Phenomena and theory related to hybrid nanofluids

### 4.1 Brownian motion

Brownian motion is a fundamental concept in nanofluid applications, crucial for enhancing both stability and thermal conductivity of hybrid nanofluids. It describes the random movement of nanoparticles, which promotes their dispersion, collision, and suspension in water. This motion ensures a uniform distribution of nanoparticles, preventing sedimentation and aggregation over time, thereby maintaining the long-term stability of hybrid nanofluids. Stability is critical for preserving the enhanced properties of the fluid over time and ensuring consistent thermal performance. Brownian motion not only facilitates uniform dispersion but also enhances heat transmission between nanoparticles, thereby increasing the overall heat transfer rate and improving thermal conductivity. Moreover, microscopic mixing induced by Brownian motion generates localized convection currents, which further enhances convective heat transfer by redistributing heat from hotter to cooler regions. The interaction between nanoparticles and the base fluid also alters the effective viscosity of the fluid, influencing its flow characteristics and contributing to improved heat transfer efficiency.^34^ Thus, understanding and harnessing Brownian motion are essential for optimizing the thermal properties and performance of hybrid nanofluids in various applications.

Several factors influence the intensity and effectiveness of Brownian motion in hybrid nanofluids, including particle size, temperature, viscosity of the base fluid, and nanoparticle concentration. Smaller nanoparticles exhibit more pronounced Brownian motion, while higher temperatures increase the kinetic energy and, thus, enhance the nanoparticle motion. Lower viscosity of base fluids, such as water, allows for freer nanoparticle movement, accelerating Brownian motion. Conversely, higher concentrations of nanoparticles can lead to aggregation, diminishing motion and reducing nanofluid stability. The optimization of these parameters is crucial to maximize the benefits of Brownian motion, enhancing thermal conductivity, stability, and efficiency of hybrid nanofluids in applications such as heat exchangers, cooling systems, solar thermal systems, medical applications, and lubricants.

**Fig. 4:**
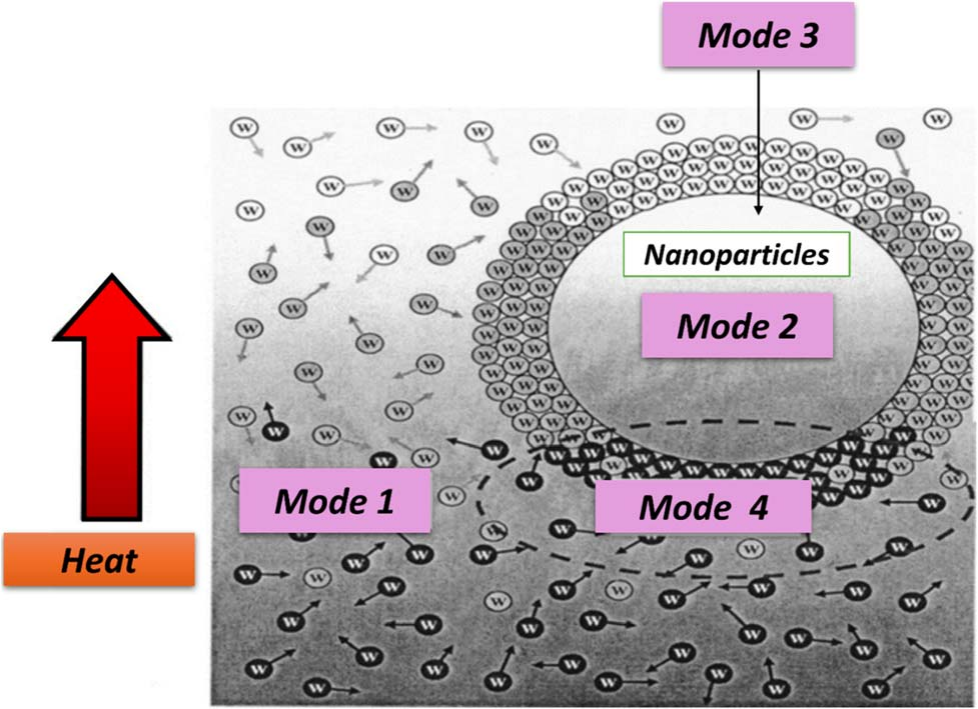
Modes of energy transport in hybrid nanofluids. Reproduced with permission.^34^ Copyright AIP Publishing 2004.

According to Brownian theory, smaller nanoparticles move faster, transferring more energy throughout the fluid. In nanofluids, Brownian motion intensifies with temperature, significantly enhancing thermal conductivity at higher temperatures by reducing the base fluid viscosity, increasing the nanoparticle movement, and improving the solid–solid heat transfer between particles, as shown in Fig. 4 (Mode 3). In other words, as the temperature of a hybrid nanofluid increases, the Brownian motion of suspended nanoparticles increases, leading to more frequent collisions and, consequently, a higher thermal conductivity.^35^ Additionally, the Brownian motion of nanoparticles helps prevent agglomeration by keeping them suspended in the base fluid. This phenomenon enhances convection-like processes, further improving thermal conductivity.^34^

Water-based hybrid nanofluids exhibit four primary modes of energy transport:

(1) Collisions between water molecules, representing the intrinsic thermal conductivity of water.

(2) Thermal diffusion within nanoparticles suspended in water.

(3) Collisions between hybrid nanoparticles due to Brownian motion.

(4) Thermal interactions between dynamic nanoparticles and water molecules, as illustrated in Fig. 4.^36^

Understanding and leveraging these energy transport modes are crucial for optimizing the thermal properties and performance of hybrid nanofluids in diverse industrial and technological applications.

### 4.2 DLVO theory

The DLVO theory, named after the four scientists who developed it in the 1940s, evaluates the physicochemical interactions between particles in colloidal systems. This theory explains why some colloidal systems agglomerate while others remain dispersed in suspension, linking the charge of particles to the distance between them. Central to this theory is the equilibrium between van der Waals attraction potential (*V*_A_) and the electrostatic repulsion potential (*V*_R_). Factors such as ion type, concentration, surface potential value, and particle size influence the stability of a system. The total potential energy (*V*_T_) is the sum of *V*_A_ and *V*_R,_ expressed as *V*_T_ = *V*_A_ + *V*_R_. The DLVO theory proposes that the interaction of energy between particles results from attractive van der Waals forces and repulsive forces from the overlap of electrical double layers around each particle. The stability in colloidal systems is achieved when repulsive forces dominate over attractive forces, thereby keeping particles dispersed. Conversely, stronger attractive forces can cause particles to aggregate, leading to destabilization.^32^ The DLVO theory plays a crucial role in understanding the behavior of colloidal particles across disciplines such as chemistry, biology, and materials science, providing insights into the conditions that determine particle dispersion or aggregation.

**Fig. 5:**
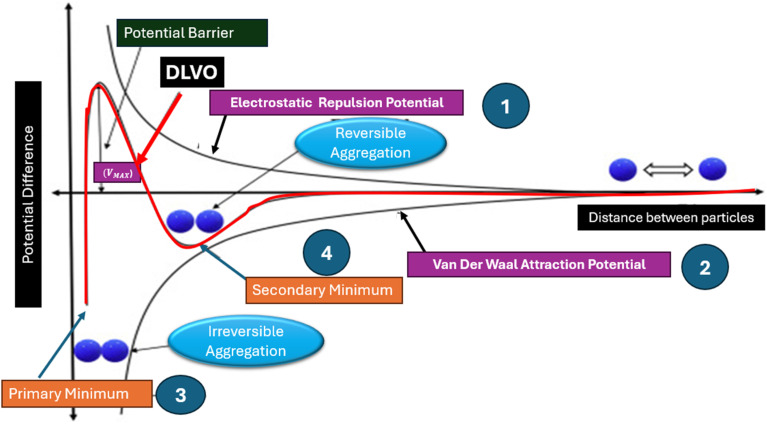
Schematic of DLVO theory.

According to Fig. 5, the *x*-axis represents the distance between particles, while the *y*-axis represents potential difference or potential energy. Several distinct scenarios can occur:

(1) Electrostatic repulsion (*V*_R_): this occurs when particles with the same charge are brought close together. The interaction potential is high, preventing agglomeration and maintaining stability.

(2) Attraction of opposing charge particles (*V*_A_): this scenario leads to an increase in van der Waals attraction potential. The total interaction potential is low, facilitating faster settling of particles.

(3) Maximum repulsion potential: when particles with the same charge are brought very close together, their repulsion potential peaks before strong bonding begin to attract them together. At high temperatures, particles can break through a strong energy barrier. Once the barrier is surpassed, attractive forces dominate over repulsive forces, causing particles to cluster irreversibly.

(4) Secondary minimum: in suspensions with a low zeta potential and a high particle loading, van der Waals potential predominates over repulsive potential, leading to a secondary minimum. This minimum distance allows weakly linked clusters to form.^37^

Vigorous agitation and ultrasonication serve as external forces to disperse clusters and restore stable nanofluid suspensions, consistent with principles outlined in the DLVO theory, as depicted in Fig. 5. Agglomeration is mitigated when the repulsive forces between particles are sufficiently strong.^38^ Significant repulsive potential is essential to prevent undesirable flocculation and coagulation. It ensures the long-term stability of nanofluids by maintaining a high energy barrier that impedes particle aggregation. This barrier plays a critical role in stabilizing nanofluids and preserving their enhanced properties over time.

**Fig. 6:**
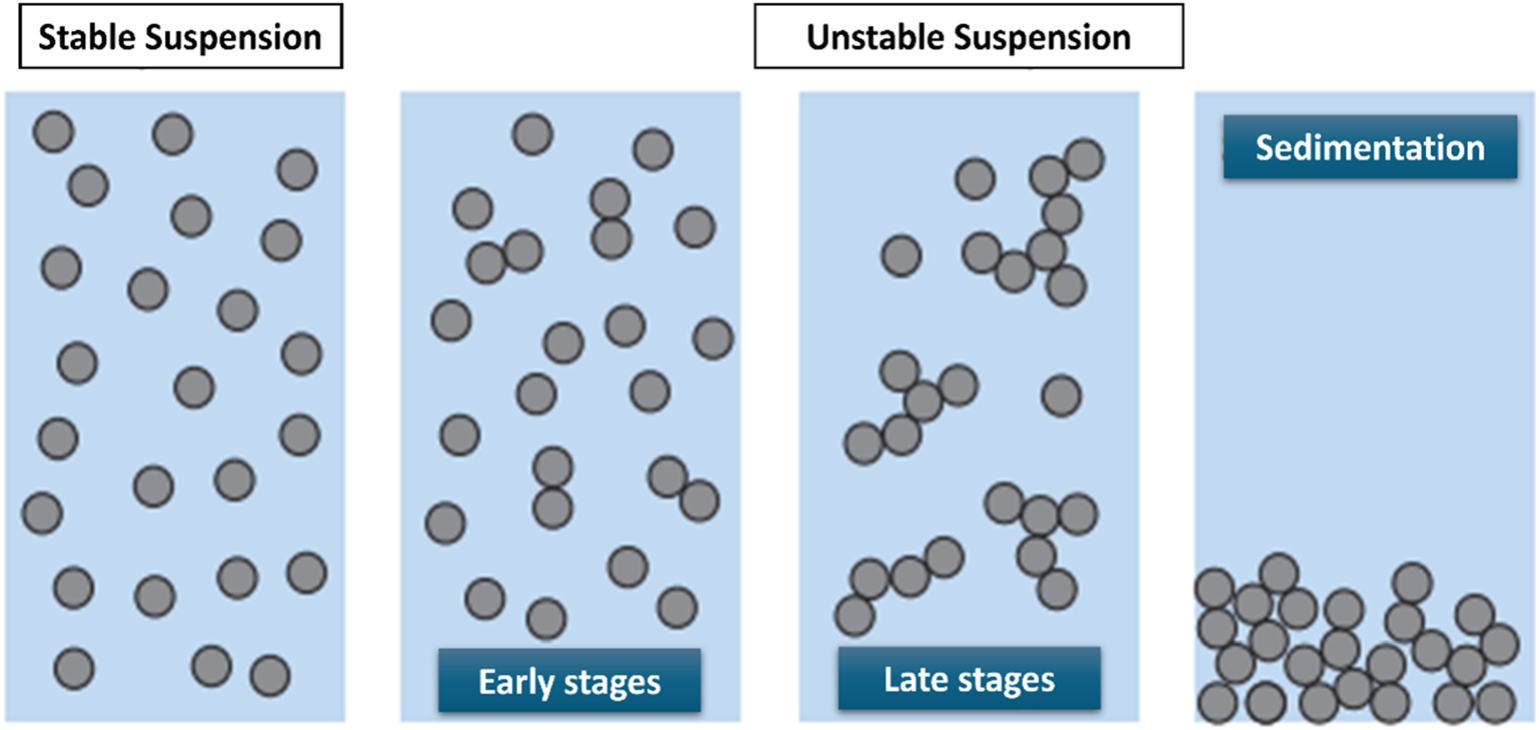
Comparison of stable and unstable suspensions. Reproduced from ref. 39 by Creative Commons Attribution 4.0 International License.

Colloidal particles interact based on the energy profile when suspended in water, as shown in Fig. 6. Particles with a strong repulsive energy profile will repel each other, resulting in a stable suspension. Conversely, when the energy profile is attractive, particles come into closer contact and stick together. As aggregation begins, particles form larger and larger flocs. As the flocs grow, sedimentation occurs, leading to the clarification of the suspension and indicating instability.^39^ The instability of the hybrid nanofluids occurs when the repulsive forces surrounding the nanoparticles drop below a certain value, where the nanoparticles begin to conjugate and slowly sediment.^40^

## 5 Stability and thermal conductivity evaluation of hybrid nanofluids

This section is divided into two parts:

(1) Stability evaluation – various testing methods are used to assess the stability of the hybrid nanofluid, including visual observation, dynamic light scattering (DLS), UV-vis spectroscopy, and morphology analysis by transmission electron microscopy (TEM).

(2) Thermal conductivity testing – the KD2 Probe is employed to measure thermal conductivity and evaluate the heat transfer efficiency of the hybrid nanofluid.

Additionally, this section highlights key precautionary steps necessary for obtaining accurate results, along with discussions on the influence of hybrid nanofluid properties on stability and thermal conductivity.

### 5.1 Visual sedimentation method and centrifugation

**Fig. 7:**
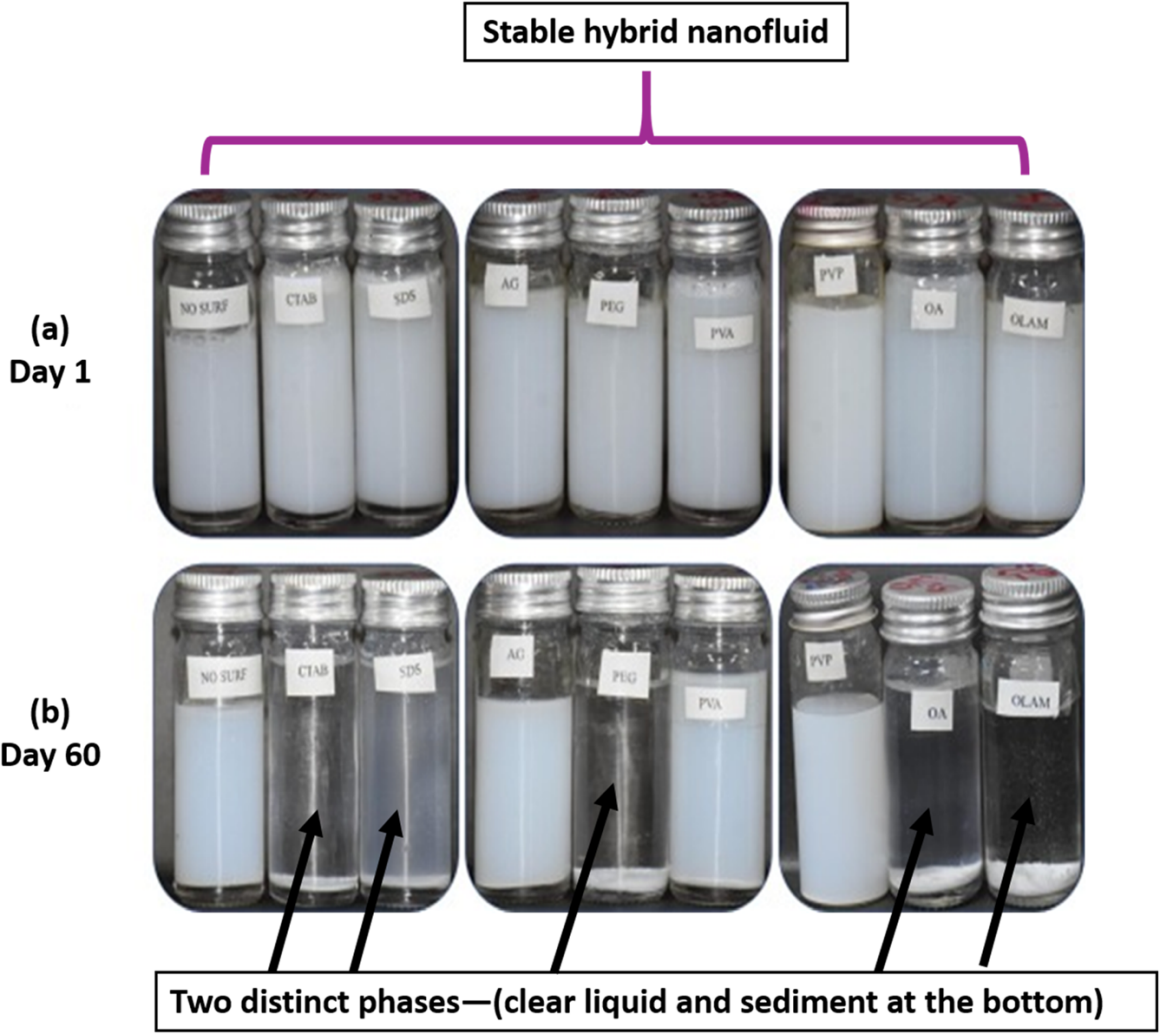
Comparison of the visual sedimentation evaluation of hybrid nanofluids at (a) day 1 and (b) day 60 using the visual sedimentation method. Reproduced with permission.^41^ Copyright Elsevier 2003.

Visual observation, also known as the photography method, is a commonly used qualitative technique for evaluating the stability of hybrid nanofluids by monitoring sedimentation. This method involves placing the hybrid nanofluid in a static environment without agitation and observing its behavior over a specific period. For example, A. Rehman *et al.*^41^ employed the visual sedimentation method to examine the effect of eight different types of surfactants on the stability of TiO_2_–Al_2_O_3_ hybrid nanofluids, as shown in Fig. 7. All surfactants were added in the same ratio (1/10th of the nanoparticle amount) to prevent excessive surfactant addition, which could reduce thermal conductivity.^41^Fig. 7(a) was captured immediately after hybrid nanofluid preparation using the two-step method, involving magnetic stirring for 30 minutes followed by sonication in a bath for 7 to 8 hours at 30 °C. Fig. 7(b) was taken after 60 days. The results indicate that polyvinylpyrrolidone (PVP) provided the highest stability among all surfactants over this period. Based on Fig. 7(b), the nanofluids containing PVP, AG, and PVA, as well as the sample without any surfactant, remained stable after 60 days. In contrast, the hybrid nanofluids with CTAB, SDS, PEG, OLAm, and OA exhibited agglomeration and sedimentation, which were visibly apparent. This instability resulted from strong van der Waals forces and the gravitational pull acting on the nanoparticles. The separation of the hybrid nanofluid into two distinct phases—a clear liquid at the top and sediment at the bottom—indicates instability.

Another alternative way to understand the stability of the nanofluid is by the centrifuge method, which can be done by a short period of time compared to the visual sedimentation method. In this method, the centrifugal force is much stronger than the normal gravitational force, which accelerates the sedimentation process. The terminal settling velocity (*V*_t_) for a spherical and smooth nanoparticle in a centrifuge can be determined considering the Stokes law regime (Rep < 1) using eqn (1):1
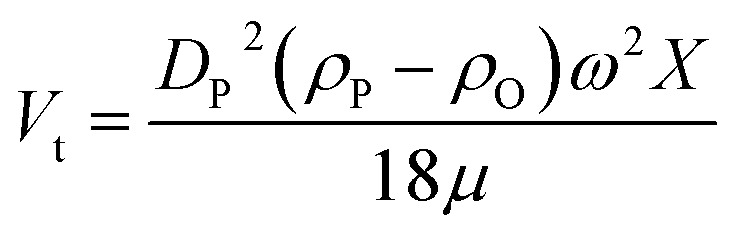
where *D*_P_, *X*, *ρ*_P_, *ρ*_O_, *ω* and *μ* represent the particle diameter, distance between the axis of rotation and the specific location of the tube inside centrifuge, density of nanoparticles, density of base fluid, angular velocity of centrifuge and viscosity of base fluid, respectively.^20^ This equation clearly indicates that the low particle size, higher viscosity of base fluid and nominal density difference between the nanoparticle and the base fluid are key to achieving higher nanofluid stability.^20^ It is also to be pointed out that lowering the particle size increases the surface energy, which, in turn, can lead to particle clustering. Higher settling velocity indicates faster settling of nanoparticles. Both sedimentation and centrifugation technique provide quantitative evaluation of stability in terms of settling time or by measuring the height of sediment layer with respect to time.

Although visual sedimentation is a useful qualitative method for observing sedimentation, it cannot predict the underlying mechanisms causing sedimentation. This phenomenon is primarily attributed to strong van der Waals forces and the gravitational settling of nanoparticles. Moreover, visual observation is less suitable for hybrid nanofluids with high nanoparticle concentrations. Another limitation is its subjectivity—observations can vary between individuals, and any disturbance of the solution may lead to inaccurate results. Additionally, hybrid nanofluids with cloudy or dark-colored solutions, such as those containing carbon nanotubes (CNTs), make it difficult to clearly observe sedimentation.^42^ Therefore, in certain cases, researchers rely on dynamic light scattering (DLS) or UV-vis spectroscopy for a more precise assessment of hybrid nanofluid stability.

### 5.2 Dynamic light scattering

**Fig. 8:**
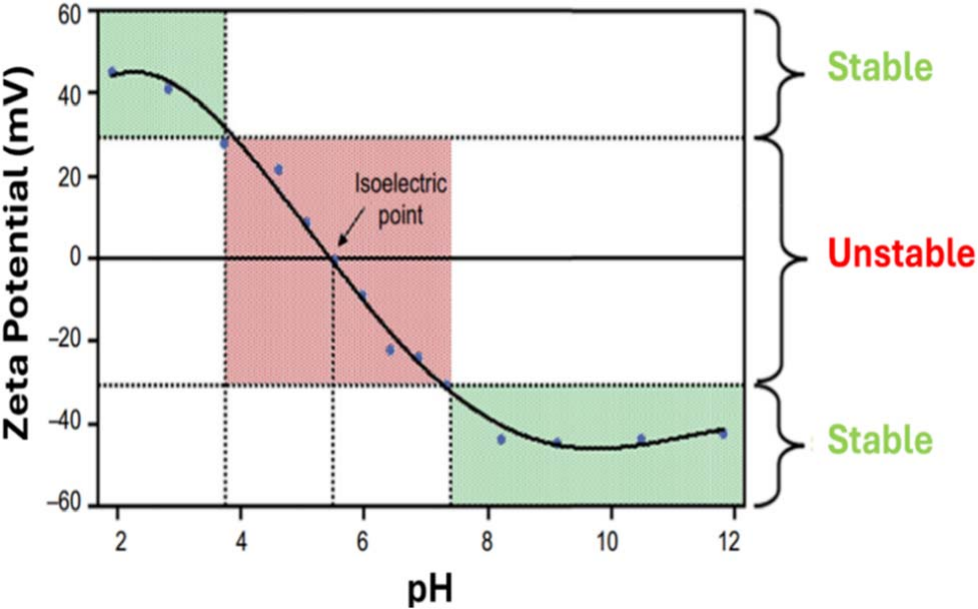
Zeta potential graph with variation in pH. Reproduced with permission.^43^ Copyright Elsevier 2016.

Dynamic light scattering (DLS) technique is another characterization method used for stability evaluation by measuring the potential difference between dispersed particles and the surrounding solution layer attached to them, which is known as zeta potential and it directly refers to the stability of the hybrid nanofluid, as shown in Table 2. The zeta potential, whether positive or negative indicates that the hybrid nanofluid is electrically charged and exhibits strong electrostatic repulsion. It is a scientific measure of electrokinetic potential in colloidal dispersions. This parameter reflects the repulsive forces between nanoparticles, with zeta potential values typically ranging from 0 to ±100 mV, as illustrated in Fig. 8. The higher the zeta potential value, the better the stability of the hybrid nanofluid. This is because the higher zeta potential value of hybrid nanofluids indicates a higher repulsive force, which prevents the nanoparticles from aggregation and formation of sediment.

**Table 2:** Zeta potential value *versus* the stability

Zeta potential value (±mV)	Stability based on observation
0–5	Rapid agglomeration
5–15	Incipient stability but settling lightly
15–30	Moderate stability
30–45	Good stability and possible settling
More than 60	Excellent stability and little settling

Babita *et al.*^17^ have reviewed the importance of stability in nanofluid preparation and ways to improve the stability of the nanofluid. According to their findings, there are a few factors to consider when analysing the zeta potential of hybrid nanofluids. First and foremost, hybrid nanofluids consisting of highly conductive nanoparticles cannot be used for zeta potential measurement because the highly conductive ions cause electrode polarization and deterioration. Additionally, the concentration of the hybrid nanofluid is a crucial factor to consider when performing zeta potential testing. Extremely high or low nanofluid concentrations can affect the zeta potential. High-concentration hybrid nanofluids produce inaccurate results because they absorb most of the incident light, reducing the intensity of scattered light. Conversely, low-concentration hybrid nanofluids produce inaccurate results because there must be a sufficient number of light-scattering elements present within the nanofluid. Besides, steric stabilization cannot be quantified using zeta potential measurement since polymer attachment to the nanoparticle surface does not change the surface potential.^20^

P. Mukesh Kumar *et al.*^44^ evaluated the stability of Al_2_O_3_–SiO_2_ water-based hybrid nanofluids at different particle weight concentrations (0.2 wt%, 0.4 wt%, and 0.6 wt%) using zeta potential analysis, UV-vis spectroscopy, and visual sedimentation methods. The zeta potential test results showed that the 0.6 wt% Al_2_O_3_–SiO_2_ hybrid nanofluid exhibited the highest zeta potential values, measuring −42.7 mV immediately after preparation and −60.7 mV after four weeks. These values indicate strong electrostatic repulsion between nanoparticles, which enhances stability. A higher zeta potential suggests improved dispersion and a longer shelf life compared to hybrid nanofluids with lower particle concentrations. Additionally, visual sedimentation analysis revealed minimal nanoparticle settlement after four weeks under a stationary condition, further confirming the stability of the nanofluid. This observation aligns with the zeta potential results, reinforcing the suitability of the 0.6 wt% hybrid nanofluid for long-term use.

A. Tiwari *et al.*^45^ conducted a study to confirm that the zeta potential is a highly effective indicator for assessing the stability of hybrid nanofluids. They synthesized a CeO_2_-MWCNT (80 : 20) water-based hybrid nanofluid using the two-step method, by varying sonication times (30, 60, 90, 120, 150, and 180 minutes) and surfactant-to-nanoparticle mixing ratios (5 : 0, 4 : 1, 3 : 2, 2 : 3, and 1 : 4). Various surfactants, including SDS, CTAB, DDC, GA, and PVP, were also used.

**Fig. 9:**
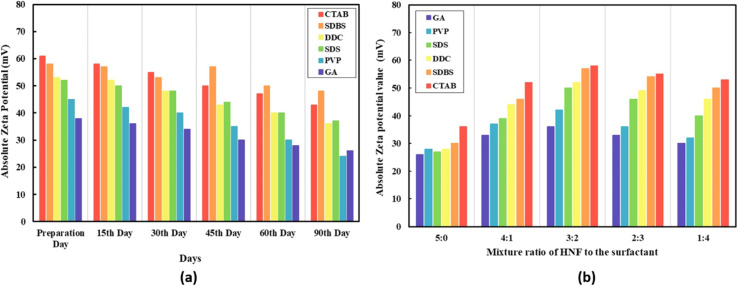
(a) Effect of different surfactants on the absolute zeta potential of the prepared (CeO_2_ + MWCNTs (80 : 20)) water-based hybrid nanofluid over 90 days from the preparation. (b) Effect of the mixture ratio of volume concentration to the surfactant on the stability of HNF (CeO_2_ + MWCNTs (80 : 20)) on the 15th day of preparation. Reproduced with permission.^45^ Copyright Elsevier 2021.

The prepared samples were analyzed at different pH levels (8–11) and storage durations (15, 30, 45, 60, and 90 days) to determine the optimal surfactant type and ratio for maintaining stability over at least 90 days. The zeta potential measurements indicated that the CTAB surfactant provided the highest stability up to 30 days. Beyond this period, the SDBS surfactant exhibited superior long-term stability when applied with a 3/2 mixing ratio and 90 minutes of sonication, as shown in Fig. 9(a). As illustrated in Fig. 9(b), an increase in surfactant concentration initially led to a significant rise in zeta potential values, enhancing the stability. However, exceeding the optimal concentration reduced both suspension stability and zeta potential. Surfactants, which contain a hydrophilic head and a hydrophobic tail, contribute to the long-term stability of hybrid nanofluids by forming a uniform suspension. This occurs as surfactant molecules adsorb onto the MWCNT walls through hydrophobic interactions, preventing nanoparticle aggregation. The absolute zeta potential of the hybrid nanofluid prepared with CTAB surfactant was 61 mV on the preparation day and 55 mV on the 30th day. In contrast, the hybrid nanofluid stabilized with SDBS exhibited zeta potential values of 57 mV and 48 mV on the 45th and 90th days, respectively. These results suggest that the anionic surfactant SDBS adsorbed onto MWCNT walls *via* hydrophobic interactions and π–π stacking (covalent bonding), enhancing the suspension stability and homogeneity even after 90 days. In conclusion, CTAB (a cationic surfactant) was found to be the most effective stabilizer for hybrid nanofluids up to 30 days, while SDBS (an anionic surfactant) provided excellent long-term stability for up to 90 days.

### 5.3 UV-vis spectrometry for stability assessment

**Fig. 10:**
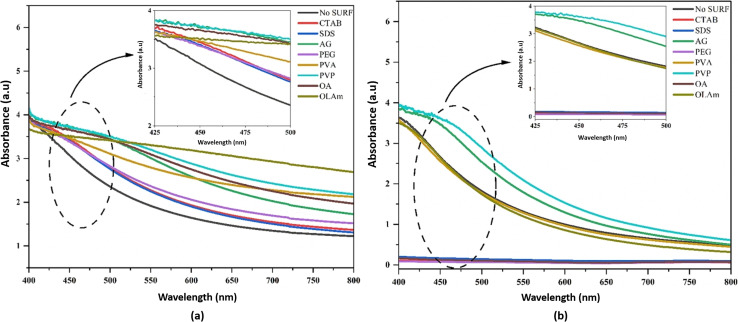
UV-vis result of hybrid nanofluids with different surfactants at (a) day 1 and (b) day 30. Reproduced with permission.^41^ Copyright Elsevier 2023.

The stability of hybrid nanofluids can also be evaluated using UV-vis spectrometry, which measures absorbance within a wavelength range of 190–1100 nm. Higher absorbance values indicate a greater distribution of nanoparticles within the nanofluid, signifying improved stability.^41^ A. Rehman *et al.*^41^ employed UV-vis spectroscopy within the 400–800 nm range to analyze the behavior of hybrid nanofluids with and without surfactants. They specifically examined absorbance peaks between 425 nm and 500 nm, as shown in Fig. 10(a) and (b). The results demonstrated that an increase in absorbance corresponded to a higher nanoparticle distribution, which directly correlates with nanofluid stability. On day 1, PVP exhibited the highest absorbance peak, followed by AG and OLAm. Meanwhile, OA, CTAB, PEG, SDS, and PVA displayed similar absorbance levels, while the hybrid nanofluid without surfactant (No SURF) showed the lowest absorbance, as illustrated in Fig. 10(a). After 60 days, UV-vis spectroscopy was repeated for all samples, with results depicted in Fig. 10(b). The findings showed that PVP retained the highest absorbance peak over time, followed by AG. PVA, OLAm, and No SURF displayed similar absorbance values, indicating moderate stability. However, CTAB, SDS, PEG, and OA exhibited no absorbance, suggesting complete nanoparticle sedimentation, as confirmed by the visual sedimentation method. Thus, according to the UV-vis spectroscopy results, PVP-, No SURF-, and AG-based hybrid nanofluids demonstrated the highest stability among all the tested surfactants.

Moreover, this method provides valuable quantitative results related to the concentration of hybrid nanofluids, as there is a direct linear relationship between nanofluid concentration and absorbance.^46^ This relationship was confirmed by Adogbeji *et al.*,^47^ who utilized UV-vis spectrometry to assess the stability of Fe_3_O_4_/TiO_2_ magnetic hybrid nanofluids based on sedimentation formation and volumetric fraction. Their findings indicated that a lower absorption rate corresponds to a decreased nanoparticle volumetric fraction, which is directly associated with a higher sedimentation formation. This observation aligns with Giwa,^48^ who reported that the absorbance increases with higher nanoparticle volume concentrations. In a 30-day study, hybrid nanofluids with volume fractions of 0.3% vol, 0.2% vol, and 0.1% vol exhibited sedimentation factors (SF) of 37.79%, 35.43%, and 31.79%, respectively. In contrast, nanofluids with lower volume fractions of 0.00625% vol, 0.0125% vol, and 0.025% vol demonstrated exceptional stability, with SF percentages of 8.89%, 9.82%, and 10.24%, respectively. The nanofluid with 0.05 vol% had an SF of 10.68%, indicating moderate stability, while higher concentrations (0.1 vol%, 0.2 vol%, and 0.3 vol%) exhibited reduced stability, suggesting the need for optimization and stabilizers at higher concentrations.^47^ The absorbance of hybrid nanofluids at different wavelengths and SF percentages was determined using eqn (2):2
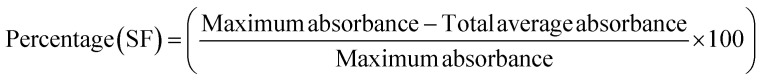


Moreover, the stability of hybrid nanofluids can be assessed by evaluating the supernatant concentration using a UV-vis spectrophotometer over time. The relative concentration (*C*/*C*_o_) is used to describe the sedimentation behavior, where a value of 1 represents an ideal, fully stable condition, while lower values indicate nanoparticle aggregation and subsequent sedimentation.^49^ An increase in absorbance corresponds to better nanoparticle dispersion within the hybrid nanofluid, which is directly associated with improved stability. However, this method has certain limitations. It is not suitable for evaluating dark-colored, water-based hybrid nanofluids or highly concentrated hybrid nanofluids. High nanoparticle concentrations can lead to excessive absorbance of incident light, diminishing the intensity of scattered light and reducing data quality.

**Fig. 11:**
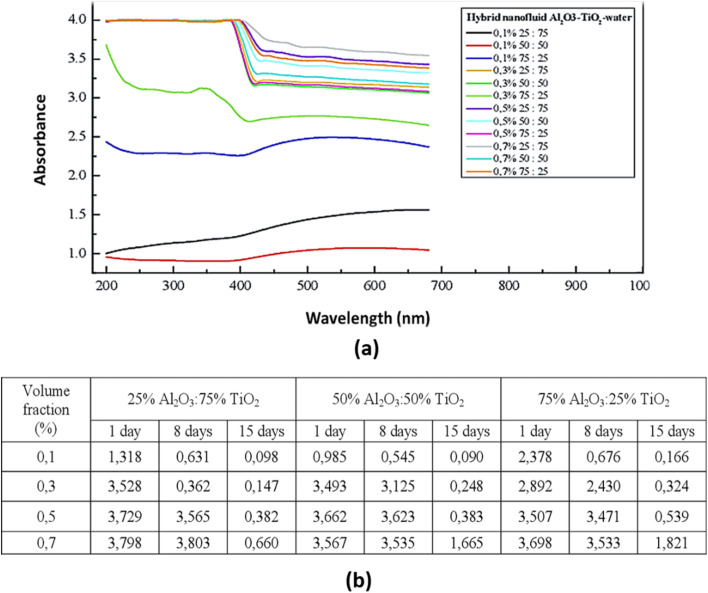
(a) UV-vis result of Al_2_O_3_–TiO_2_ water-based hybrid nanofluids, and (b) the absorbance value of hybrid nanofluids at a wavelength ranging from 200 to 680 nm. Reproduced with permission.^42^ Creative Commons Attribution 4.0 International License.

Septiadi *et al.*^42^ also utilized UV-vis spectroscopy (Fig. 11(a)) to evaluate the stability of water-based Al_2_O_3_–TiO_2_ nanofluids. In this study, Al_2_O_3_–TiO_2_ nanoparticles were dispersed in water at volume fractions of 0.1%, 0.3%, 0.5%, and 0.7%, with Al_2_O_3_–TiO_2_ ratios of 75% : 25%, 50% : 50%, and 25% : 75%, respectively. The synthesis process involved 30 minutes of magnetic stirring. As shown in Fig. 11(b), nanofluids with 75% Al_2_O_3_ nanoparticles exhibited higher absorbance values, indicating better stability than hybrid nanofluids with higher TiO_2_ concentrations. This improved stability is attributed to the prolonged Brownian motion of Al_2_O_3_ nanoparticles and their lower density, which reduces sedimentation. Additionally, at higher volume fractions, stronger frictional forces between nanoparticles slow down agglomeration, allowing the particles to remain suspended in the base fluid for a longer duration, thereby enhancing the nanofluid stability.

### 5.4 Morphology characterization

**Fig. 12:**
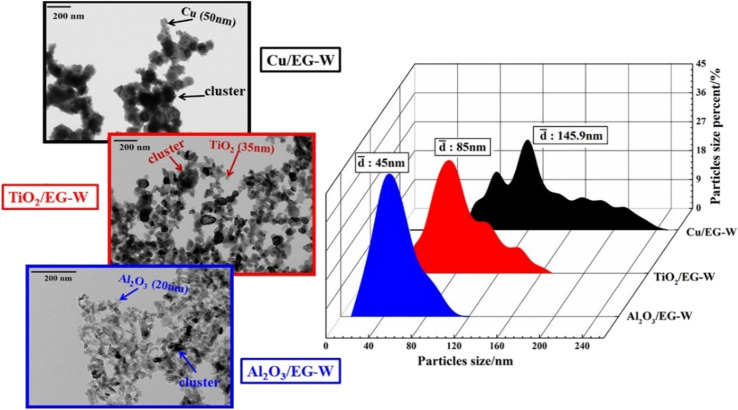
Stability evaluation of mono-nanofluids using TEM. Reproduced with permission.^51^ Copyright Elsevier 2020.

Another method for determining the stability of hybrid nanofluids is morphology analysis using a transmission electron microscope (TEM). Vicki *et al.*^50^ utilized TEM to examine the morphology of TiO_2_ and Al_2_O_3_ nanoparticles in a hybrid nanofluid, revealing that both nanoparticles exhibited a spherical shape. Similarly, Giwa *et al.*^48^ demonstrated that TEM is effective in evaluating the morphology and dispersion of MWCNT-Fe_2_O_3_ hybrid nanofluids. This high-resolution technique allows for the visualization of aggregated nanoparticles based on their size, as shown in Fig. 12. In this study, TEM was used to assess the stability of a water-based hybrid nanofluid by analyzing cluster sizes using the ImageJ software.^51^ The measured cluster sizes were approximately 45 nm for Al_2_O_3_/EG–W, 85 nm for TiO_2_/EG–W, and 145.9 nm for Cu/EG–W. The study concluded that smaller cluster sizes enhance the stability and thermal properties, as they enable faster nanoparticle movement and more efficient energy exchange with the base fluid. In contrast, larger clusters lead to greater agglomeration, reducing Brownian motion and ultimately lowering the stability and thermal conductivity.

**Fig. 13:**
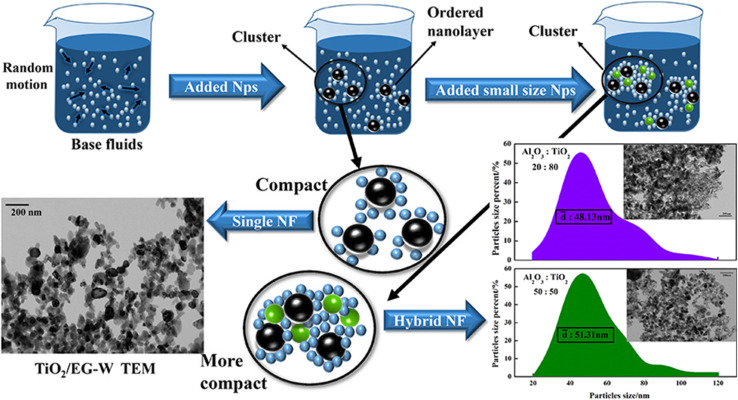
Synergistic mechanism of thermal conductivity enhancement. Reproduced with permission.^51^ Copyright Elsevier 2020.

Fig. 13 illustrates the synergistic approach to enhancing the thermal conductivity, which involves dispersing nanoparticles of varying sizes into the base fluid. This process leads to the formation of an ordered arrangement of liquid molecules around the nanoparticles and the development of a compact solid–liquid interface, thereby facilitating efficient heat transfer and improving thermal conductivity. van der Waals forces cause nanoparticles to aggregate into clusters, forming larger nanolayered structures. These layers create particle-dense regions that exhibit reduced heat resistance, functioning as thermal bridges that link liquid molecules and nanoparticles. This intermediate physical state, analogous to the solid–liquid interface, effectively enhances the thermal conductivity of nanofluids. However, van der Waals forces can have both positive and negative effects on thermal conductivity. Initially, they promote cluster formation, which accelerates heat transfer within these structures, significantly improving thermal conductivity. Over time, however, as the cluster mass increases, the enhancement diminishes due to increased sedimentation. To counteract this effect, physical vibrations can be applied to break down sedimentation and maintain the stability and effectiveness of the nanofluid.

### 5.5 Thermal conductivity evaluation of hybrid nanofluids

In addition to stability evaluation, thermal conductivity is a crucial parameter for assessing the thermal efficiency of hybrid nanofluids. It is most commonly measured using a KD2 Probe digital thermal analyzer, which consists of a single-needle sensor and a readout unit.^52^ The single-needle sensor (1.30 mm in diameter, 60 mm in length) must be at least 90% immersed in the hybrid nanofluid to ensure accurate measurements. This sensor is equipped with a heating element and a thermoresistor.^53^ For precise thermal conductivity measurements, the probe must be inserted vertically into the nanofluid to minimize the convection effects. Additionally, the KD2 meter should be calibrated with deionized water before each measurement set. During testing, the probe generates heat within the fluid sample, and the thermoresistor detects the resulting temperature changes, which are then recorded. The sensor operates effectively within a temperature range of −50 °C to 100 °C. However, at elevated temperatures, measurement errors may occur due to the decreased viscosity of the fluid, which can enhance convective heat transfer and affect thermal conductivity readings. To mitigate this issue, experiments are conducted using a water bath to maintain consistent temperature control. Proper calibration with pure water before each set of experiments is essential to ensure reliable and accurate results.

The thermal conductivity of nanofluids highly depends on the solid volume and also temperature. This statement is agreed by Hemmat Esfe *et al.*,^54^ who studied the effect of the volume fraction of CNTs/Al_2_O_3_ (0.02, 0.04, 0.1, 0.2, 0.4, 0.8 and 1.0%) and temperature (303, 314, 323 and 332 K) on the thermal conductivity of CNTs/Al_2_O_3_ water-based nanofluids. Besides that, Devarajan *et al.*^55^ also observed that the thermal conductivity of CNT/Al_2_O_3_ nanofluids increases with temperature. Specifically, the relative thermal conductivity of CNT and CNT/Al_2_O_3_ nanofluids increased from 1.02 to 1.27 and from 1.11 to 1.29, respectively, over a temperature range of 40–60 °C. With a nanoparticle concentration of *φ* = 0.1% [CNT (50%)/Al₂O₃ (50%)] in the base fluid, the thermal conductivity showed a 29% increase compared to the base fluid and a 27% increase compared to the CNT-based nanofluid. This is because temperature variations affect the Brownian motion of nanoparticles, significantly improving the thermal conductivity, especially at higher nanoparticle volume fractions and temperatures. This improvement occurs because nanoparticles, with their higher thermal conductivity than that of the base fluid, transfer kinetic energy through collisions and interactions. Consequently, hybrid nanofluids, which incorporate various nanoparticles, often exhibit enhanced thermal conductivity. Nanoscale particles contribute to this effect through Brownian motion, facilitating energy exchange *via* heat conduction between moving nanoparticles and heat convection between nanoparticles and the base fluid. Moreover, several theories have been presented by a number of researchers in order to explain the thermal conductivity enhancement, such as heat transfer due to the Brownian motion of particles in nanofluids and the formation of interfacial layer around the nanoparticles.^56^

Next, it is well known that graphene oxide (GO) is a nanoparticle with high surface area, high thermal conductivity, and low density, but it is expensive compared to other nanoparticles. Therefore, Taherialekouhi *et al.*^57^ selected Al_2_O_3_ nanoparticles, which have high chemical stability, thermal conductivity, and a low coefficient of thermal expansion, and are significantly cheaper than GO for water-based nanofluids. This also reduces the viscosity and preparation costs of the nanofluid. The study highlighted that thermal conductivity increased with the increase in temperature, with the highest thermal conductivity coefficient increasing by about 33.9% at 50 °C at 1% volume fraction of nanoparticles. As the temperature of the nanofluid rises, the base fluid molecules experience higher motion, vibration, and velocity, colliding more frequently with the nanoparticles. Although the number of collisions is limited due to the smaller mass of the fluid molecules, the number of encounters is infinitely high. Brownian motion and micro-scale displacement also contribute to increased thermal energy. In other words, rising temperature weakens molecular interactions in fluid layers, leading to increased nanoparticle mobility and collisions, ultimately increasing the thermal conductivity of the nanofluid.

## 6 Stability and thermal conductivity improvement of water-based hybrid nanofluids

This section is divided into two parts: chemical alterations and physical alterations, as shown in Table 4, both of which may enhance the thermal conductivity and stability of hybrid nanofluids.

### 6.1 Chemical modification

#### 6.1.1 Incorporation of surfactants

Metal and carbon-based nanoparticles are typically hydrophobic, which makes them unsuitable for practical applications due to their lack of stability in base fluids over extended periods. To enhance the stability of nanofluids, surfactants are commonly used to reduce the surface tension of the fluid and improve the particle dispersibility.^58^ Surfactants, which are also known as surface-active agents, interfacial-active agents, or dispersants, are chemicals or compounds that significantly reduce the interfacial tension between two liquids, a liquid and a gas, or a liquid and a solid.^59^ It helps to maintain the suspension of nanoparticles in base fluids. Various types of surfactants have been proven to effectively homogenize nanoparticles in base fluids.^58^ Moreover, surfactants are also widely used to modify the solid/liquid interface or to prevent the aggregation of nanoparticles.^58^ The most widely used surfactants for water-based nanofluid applications are sodium dodecylbenzene sulfonate (SDBS), cetyltrimethylammonium bromide (CTAB), gum arabic (GA), polyvinyl pyrrolidone (PVP), and sodium dodecyl sulfate (SDS), as shown in Table 3.

**Table 3:** Types of surfactants most used in water-based hybrid nanofluids and their chemical structure and classification

No.	Type of surfactant	Structure of surfactant	Classification of surfactant	Benefit
1	SDBS	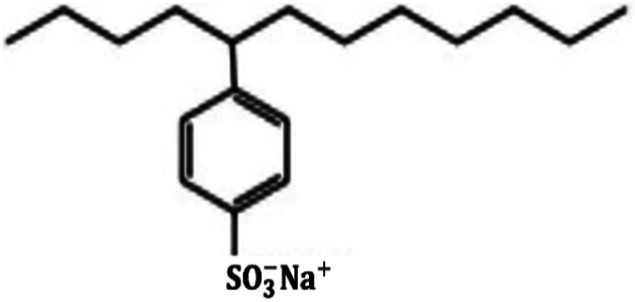	Anionic surfactant	Positively impact the stability of hybrid nanofluids^40^
2	CTAB	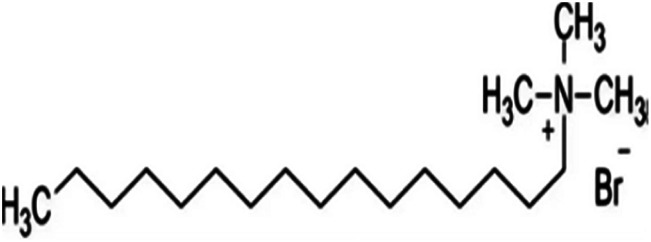	Cationic surfactant	CTAB surfactant demonstrated a superior stabilizing effect^60^
3	GA	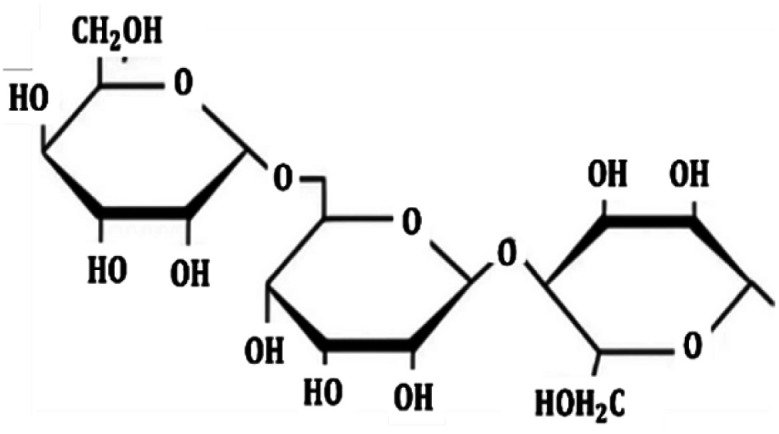	Non-ionic surfactant	Alumina nanoparticle/withstand more than 6 months^61^
				19
4	PVP	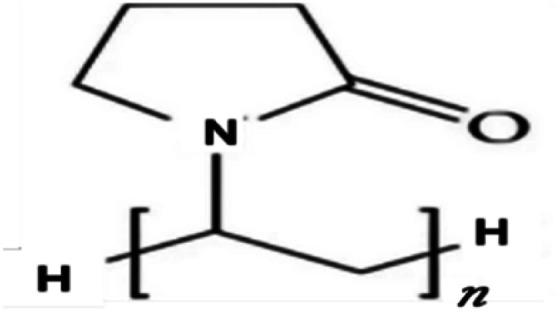	Non-ionic surfactant (steric stabilizer)	Withstand more than 6 months^19^
5	SDS	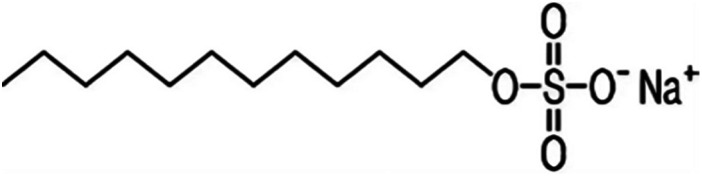	Anionic surfactant	Cost-effective surfactant^62^
6	Triton X-100	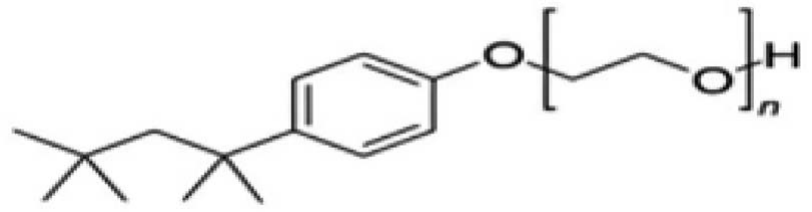	Non-ionic surfactant	Suitable for non-metallic materials (nanocellulose)^49^

**Table 4:** List of chemical and physical alterations that can be performed to improve the stability and thermal conductivity of water-based hybrid nanofluids

Chemical modification	Physical modification
• Incorporation of surfactant	• Optimization of the volume fraction of hybrid nanocomposites
• Surface modification	• Selection of the suitable type and size of nanoparticles
	• Optimum ultrasonication time
	• pH variation

**Fig. 14:**
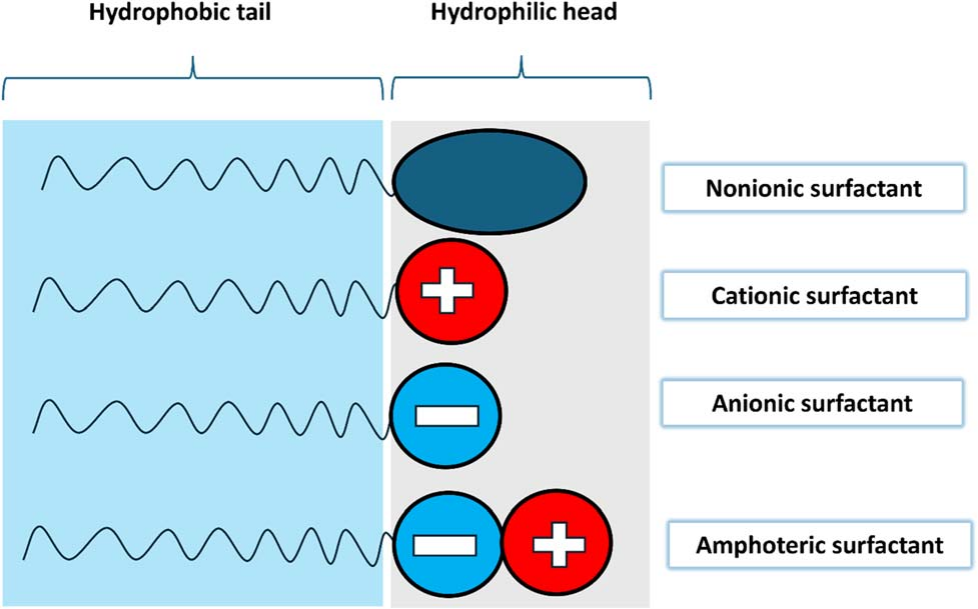
Structure and different classifications of surfactants.

Surfactants are classified into four types based on their head composition: non-ionic (no charge), anionic (negative charge), cationic (positive charge), and zwitterionic (having both positive and negative charges),^63^ as shown in Fig. 14. Their chemical structure typically includes a hydrophilic head and a hydrophobic tail, which helps nanoparticles remain dispersed in water over time. The addition of surfactants in base fluids is to improve the hydrophilic characteristics of nanoparticles and to homogenize them, thereby increasing the stability of nanofluids. The effect of a surfactant can vary depending on its type, as it can convert nanoparticles from hydrophilic to hydrophobic or *vice versa*. This capability allows surfactants to adjust the hydrophilic/hydrophobic properties of nanoparticles according to specific needs.^52^ Surfactants are a cost-effective and widely used method for stabilizing nanofluids by altering their surface properties. The incorporation of surfactants also affects the rheology of colloidal suspensions, as surfactants can increase the viscosity of the fluid. They induce steric or electrostatic repulsion between particles, reducing interparticle attraction, preventing agglomeration, and enhancing nanoparticle dispersion, which improves stability. However, exceeding the optimal concentration of surfactant can decrease thermal conductivity. A higher surfactant concentration can fill a larger volume in the fluid, increasing its viscosity and impeding heat transfer across the surface of the nanoparticles.^64^

Wai *et al.*^40^ studied the effect of surfactants (SDBS) on the stability of the ZnO–CuO/water hybrid nanofluids. It was clear that the addition of SDBS in the hybrid nanofluid increased the zeta potential, which was considered to be more stable than without the addition of surfactant. This is due to the surface charge of the nanoparticles being retained due to the adsorption of SDBS, which acts as an ionic surfactant. This created a counter balance van der Waals force of attraction and provided electrostatic repulsive force acting on two similarly charged nanoparticles. In addition, the presence of SDBS aids in a proper dispersion process and helps increase the stability, and thus, the sedimentation of nanoparticles is not observed on the surface of the heated plate. Besides the stability improvement, the addition of surfactants also helps to increase the thermal conductivity of the hybrid nanofluid as compared to the base fluid and the hybrid nanofluid without the presence of surfactants. This shows that the presence of nanoparticles promoted micro-convection in the fluid, which, in turn, enhanced the heat transfer rate. The stability of the hybrid nanofluids is crucial in ensuring that the heat received is well dispersed among the fluids for a better convection rate. The instability of the hybrid nanofluids due to agglomeration shows that the hybrid nanofluids performed worse than the base fluid at a temperature of 20–30 °C, but as the temperature increases to 40 °C, the presence of metal-oxide nanoparticles aided in improving the heat transfer rate as compared to water but still under-performed as compared to stable hybrid nanofluids with the surfactant SDBS. In this study, a high thermal conductivity value is important, as it carries a better ability to disperse heat at a better rate and thus improves the jet impinging cooling performance.

Next, Asadi *et al.*^60^ investigated the use of various surfactants—CTAB, SDS, and oleic acid (OA)—in Mg(OH)_2_ water-based nanofluids. The results demonstrated that surfactants significantly enhance the stability of water-based nanofluids. Among them, CTAB was identified as the most effective surfactant, based on its superior zeta potential and its impact on stability after 30 minutes of sonication. Here, sonication serves the purpose of dissolving the surfactant in water, breaking down the van der Waals interactions between nanoparticles.^60^ Besides that, Almanassra *et al.*^19^ have studied the effect of different types of surfactants such as GA, PVP and SDS on the stability and thermo-physical properties of CNT/water-based nanofluids. The stability of nanofluids was noticed to withstand more than 6 months with the GA and PVP surfactants at CNT-to-surfactant ratios of 1/05 and 1/1 as optimum values, respectively. Additionally, Harun *et al.*^49^ demonstrated that surfactants such as Triton X-100 can enhance the stability of nanocellulose–water nanofluids. Therefore, selecting an appropriate surfactant and the correct ratio with nanoparticles is crucial, as it can modify electrostatic repulsion and balance the van der Waals forces of attraction. U. S. Shenoy and A. N. Shetty also proved that surfactants such as PVP can act as stabilizing agents, rendering the nanofluid stable for 9 weeks.^65^

Alumina nanoparticles are one of the widely used nanoparticles in the nanofluid application to increase the thermal conductivity of the nanofluid. However, there is still an issue with instability. As a result, some researchers used different types of surfactants such as SDBS, SDS, and CTAB to increase the stability of alumina-based nanofluids. Same goes to Nurdin *et al.*,^61^ who used GA as a surfactant to increase the stability of alumina/water nanofluids at different ratios of alumina nanoparticles and surfactant concentrations. The results showed that decreasing the nanoparticle volume fraction and increasing the GA concentration increased the stability of water-based alumina nanoparticle suspensions, resulting in a narrow distribution particle size with smaller hydrodynamic diameters of the particles. The smallest particle size recorded was 164.6 nm at 0.5% GA concentration and the highest zeta potential was recorded at −36.0 mV compared to the zeta potential value of −25 mV at 0.1% GA concentration. As the GA concentration increased, the values of the zeta potential increased correspondingly. Besides the GA concentration, the particle volume fraction of alumina has effect on the stability of nanofluids. It can be confirmed that the zeta potentials at 0.1% and 0.5% particle volume fractions are −25.7 mV and −21.3 mV, respectively. This leads to the conclusion that the suspension is more stable at lower particle volume fractions. A lower particle volume fraction means fewer particles are present, reducing the chances of particle agglomeration. This scenario promotes greater stability in the suspension. Therefore, it is plausible to assume that GA could be effectively used to stabilize water-based alumina nanoparticle suspensions at low particle volume fractions, provided that the GA content is high.^61,66^ Sodium dodecyl sulfate (SDS) is a cost-effective surfactant often employed in large-scale industrial applications. It helps maintain a uniform distribution of nanoparticles in the base fluid, prevents agglomeration, and stabilizes and disperses the nanoparticles. Additionally, SDS can improve the heat transfer performance of the nanofluid.^62^

Studies showed that SDS, CTAB and PVP can effectively reduce the aggregation from different mechanisms such as steric repulsion and electrostatic (charge) repulsion.^67^ In the latter mechanism, two nanoparticles with the same charge repel each other. Meanwhile, the steric stabilization can produce an additional steric repulsive force by adsorbing it onto the surface of nanoparticles.

#### 6.1.2 Surface modification

The stability of nanofluids is a critical challenge that must be addressed to maintain the optimal thermophysical properties of hybrid nanofluids. Aggregation can hinder uniform dispersion and lead to sediment formation over time, ultimately affecting performance in long-term applications. Various strategies have been explored to overcome these challenges, with chemical modification being one of the most effective approaches.^68^ For instance, Mandev *et al.*^69^ demonstrated that the stability of water-based hybrid nanofluids significantly improves with the addition of modified Fe_3_O_4_ nanoparticles. Characterization tests confirmed that among all the analyzed samples, the Fe_3_O_4_@SiO_2_-mix-(CH_2_)_3_Cl@imidazole-water nanofluid exhibited superior stability, making it a promising candidate for enhanced long-term performance.

Abbasi *et al.*^70^ found that the surface modification of multi-walled carbon nanotubes (MWCNTs) significantly impacts the stability and thermal conductivity of MWCNTs/γ-Al_2_O_3_ hybrid nanofluids. Their results revealed that the presence of carboxylic acid groups (–COOH) on the MWCNT surface plays a crucial role in determining both thermal conductivity and stability. Three types of hybrid nanofluids were prepared in the study:

(1) A nanofluid containing MWCNTs functionalized using 65% nitric acid (S50).

(2) A nanofluid containing MWCNTs functionalized using a mixture of 98% sulfuric acid and 65% nitric acid in 3/1 (v/v) ratio (SF50).

(3) A nanofluid containing unfunctionalized MWCNTs (PS50).

**Fig. 15:**
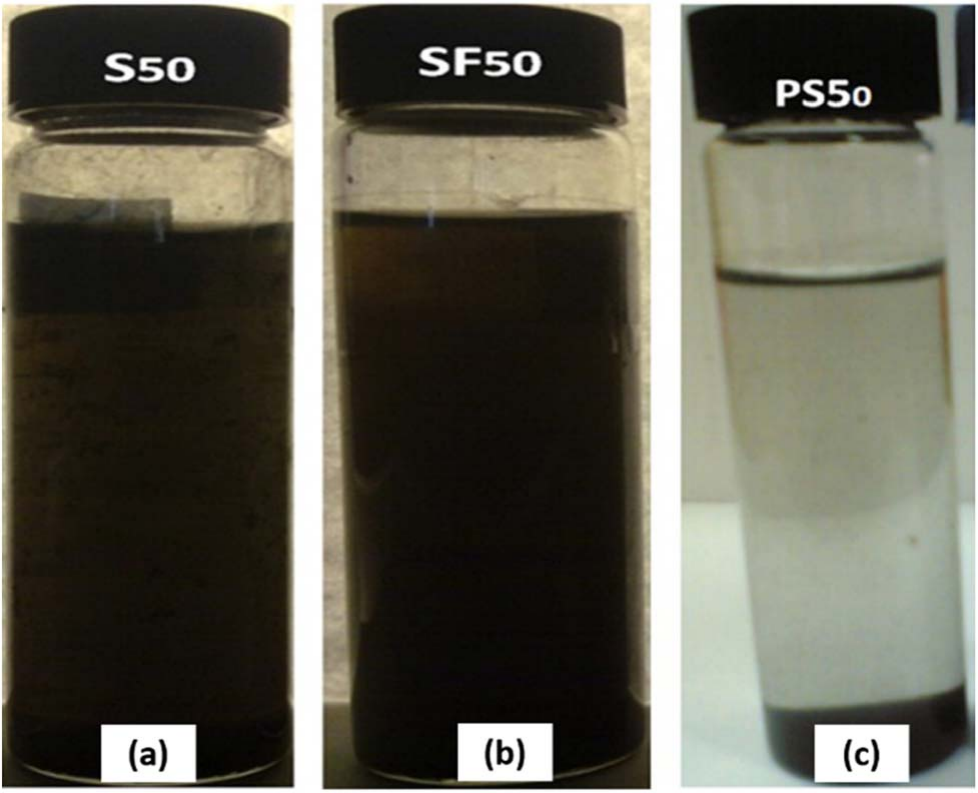
Visual observation test for the functionalized MWCNT hybrid nanofluid: (a) S 50, (b) SF50 and (c) hybrid nanofluid with unfunctionalized MWCNTs (PS 50). Reproduced with permission.^70^ Copyright Elsevier 2013.

The results indicated that hybrid nanofluids with a lower concentration of carboxylic acid groups on the MWCNT surface exhibited better stability and higher thermal conductivity than those with a higher concentration of –COOH groups, which showed reduced stability and thermal performance. Specifically, the hybrid nanofluid with a lower –COOH concentration improved thermal conductivity by up to 14.75% at a 1% volume fraction in a GA-based nanofluid, compared to unmodified carbon nanotubes. Additionally, zeta potential analysis was performed to evaluate the dispersion stability after 45 days at neutral pH values. The zeta potential of the hybrid nanofluids with functionalized MWCNTs was −22.3 mV (S50) and −22.1 mV (SF50), indicating stability over several weeks. In contrast, the zeta potential of the hybrid nanofluid with unfunctionalized MWCNTs was −16.8 mV, confirming that surface modification improves nanofluid stability. This improvement is attributed to the functional groups in oxidized MWCNTs, which reduce attractive van der Waals interactions, promoting better dispersion in the base fluid. Visual observations further confirmed that functionalized MWCNTs mitigated van der Waals attractions, leading to a more stable hybrid nanofluid. Conversely, the hybrid nanofluid with unfunctionalized MWCNTs exhibited a high tendency for aggregation and sedimentation at the bottom, even after prolonged sonication, as shown in Fig. 15. The study also highlighted that the enhancement in thermal conductivity is directly related to the dispersion of nanoparticles in the base fluid, with the overall behavior of particle–water interactions being dependent on the properties of the particle surface. Ultimately, they concluded that the thermal conductivity of hybrid nanofluids is strongly influenced by their stability.^70^

**Fig. 16:**
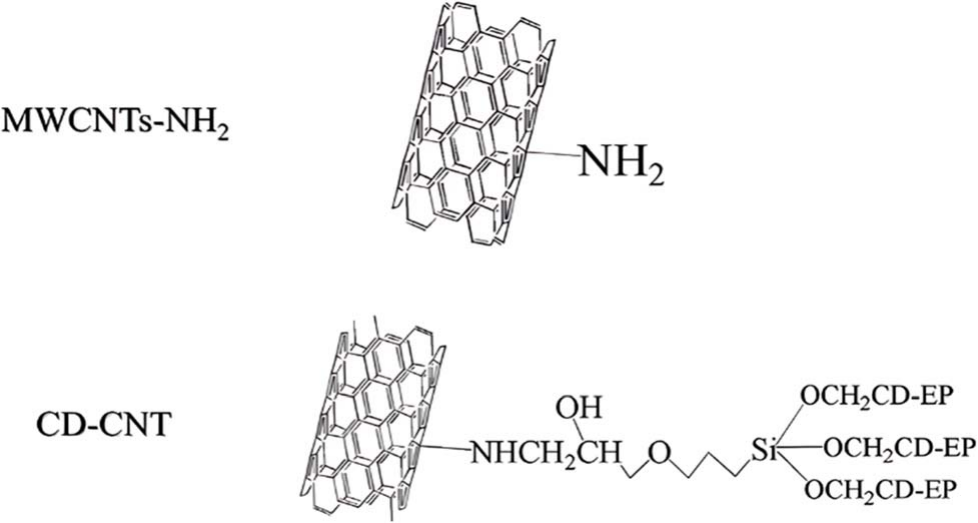
MWCNTs-NH_2_ before and after modification. Reproduced with permission.^71^ Copyright Elsevier 2020.

Li *et al.*^71^ demonstrated that modification and preparation methods can significantly enhance the thermal conductivity and stability of carbon-based nanofluids. They synthesized surface-modified multi-walled carbon nanotubes (CD-CNTs) using β-cyclodextrin and incorporated a high concentration of modified MWCNTs (5 wt%) to produce a water-based hybrid nanofluid. Typically, studies on MWCNTs use lower nanoparticle concentrations due to potential instability and increased viscosity at higher concentrations, which can hinder practical applications. However, Li *et al.* successfully utilized a high nanoparticle concentration by modifying the MWCNTs' surface. β-Cyclodextrin, with its numerous hydroxyl (–OH) groups, as shown in Fig. 16, improved the hydrophilicity of MWCNTs, enhancing their dispersion in the base fluid. This modification reduced agglomeration and mitigated the entanglement of carbon nanotubes, leading to improved stability. The thermal conductivity and enhancement values of the nanofluids increased with mass concentration at room temperature. Notably, nanofluids with a higher particle loading (5 wt%) exhibited a significant improvement in thermal conductivity compared to those with lower concentrations. A remarkable 31.99% enhancement in thermal conductivity was observed at 1 wt% compared to the base fluid. At 5 wt%, the thermal conductivity of CD-CNT nanofluids reached its peak value of 1.013 W (m^−1^ K^−1^), with an enhancement of 69.68%. This result highlights the effectiveness of surface modification in improving the thermal conductivity, colloidal stability, and overall heat transfer efficiency of MWCNT-based nanofluids, all while maintaining minimal viscosity increases.

Graphene, a form of carbon, exhibits exceptional electrical, thermal, mechanical, and catalytic properties. However, these superior characteristics are primarily associated with single-layer graphene. The solvent-phase oxidation–reduction method is widely used to synthesize single-layer graphene due to its ability to functionalize the graphene surface for various applications.

A major drawback of this method is the tendency of graphene layers to restack during the reduction of graphene oxide (GO), driven by van der Waals forces. This restacking leads to the formation of a graphitic structure, which significantly degrades the unique properties of single-layer graphene. To address this issue, the surface modification of GO before reduction is crucial. The chemical functionalization of graphene with foreign stabilizers—such as small organic molecules, low-molecular-weight polymers, or biomolecules—can enhance its dispersibility in both organic and aqueous solvents. Functional groups attached to the graphene surface *via* covalent or non-covalent bonds improve its hydrophilic or organophilic nature, facilitating better dispersion in the chosen medium. Due to its inherent hydrophobicity, graphene has a strong tendency to agglomerate in water. To enhance the stability and performance of graphene nanofluids, researchers have focused on functionalizing the graphene surface. Surface modification has proven to be an effective strategy for maintaining both the thermal conductivity and long-term stability of graphene in nanofluid applications.

**Fig. 17:**
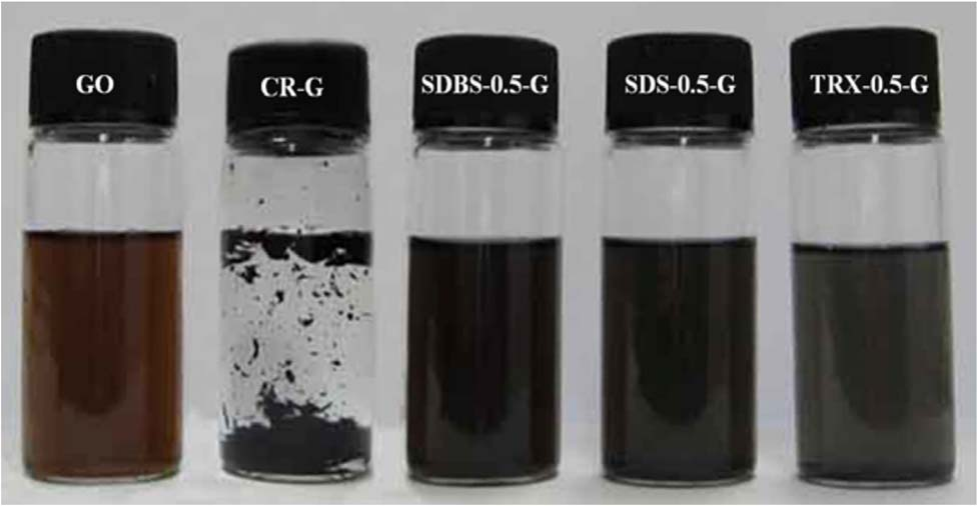
Digital photographs of water dispersion of pure GO, CR-G, SDBS-0.5-G, SDS-0.5-G and TRX-0.5-G. Reproduced with permission.^72^ Copyright Elsevier 2013.

For example, Uddin *et al.*^72^ synthesized graphene oxide (GO) from natural graphite using a modified Hummers' method. In their study, they investigated the effect of surfactant concentration on the dispersion stability of functionalized graphene compared to chemically reduced graphene (CR-G). They used both ionic surfactants—sodium dodecyl benzene sulfonate (SDBS) and sodium dodecyl sulfate (SDS)—as well as the non-ionic surfactant Triton X-100 (TRX). The functionalized graphene samples were designated as SDBS-*S*-G, SDS-*S*-G, and TRX-*S*-G, where “*S*” represents the surfactant-to-GO ratio (*S* g surfactant : 1 g GO). Fig. 17 presents the digital photographs of aqueous dispersions of pure GO, CR-G, SDBS-0.5-G, SDS-0.5-G, and TRX-0.5-G. The images clearly show that CR-G exhibited poor stability in water compared to pure GO and functionalized graphene, which remained well dispersed. The maximum dispersibilities of SDBS-0.5-G, SDS-0.5-G, and TRX-0.5-G were recorded as 1.5, 1.2, and 1.4 mg mL^−1^, respectively, whereas pure GO exhibited a higher dispersibility of 3.5 mg mL^−1^ in water. The superior dispersibility of GO was attributed to the presence of oxygen-containing functional groups on its surface. Upon reduction, these oxygen functionalities were removed, leading to a decline in dispersion stability. The dispersion characteristics of SDBS-*S*-G, SDS-*S*-G, and TRX-*S*-G were influenced by the presence of small amounts of hydroxyl (–OH) and sulfonate (–SO_3_) groups from the surfactants, which contributed to improved stability. Notably, the aqueous dispersions of functionalized graphene remained stable for over two months at room temperature, demonstrating the effectiveness of surfactant-assisted functionalization in maintaining dispersion stability.

**Fig. 18:**
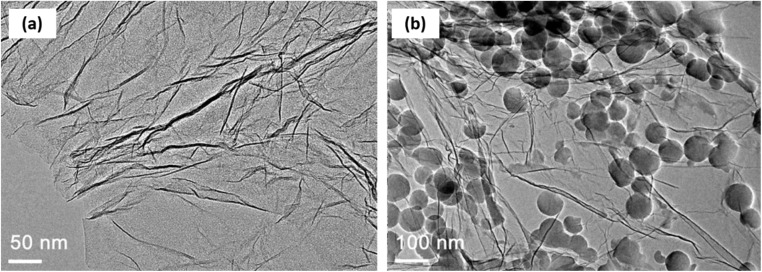
TEM images of naked graphene (a) and graphene/SiO_2_ (b) nanomaterials. Reproduced with permission.^73^ Copyright Elsevier 2014.

Li *et al.*^73^ synthesized SiO_2_-coated graphene using a chemical liquid deposition method with tetraethyl orthosilicate (TEOS). Coating an inorganic layer onto a nanomaterial surface is an effective strategy for enhancing properties such as chemical stability, colloidal stability, and biocompatibility. SiO_2_ is a commonly used coating material due to its excellent characteristics including hydrophilicity, chemical inertness, biocompatibility, and ultraviolet resistance. These properties ensure that the chemical and physical integrity of the coated material is preserved. Additionally, the well-established synthesis technology of SiO_2_ allows for its easy deposition on various materials including graphene oxide.^74^ In this study, SiO_2_ particles were successfully coated onto the graphene surface, as confirmed by TEM imaging (Fig. 18(a)). Fig. 18(b) reveals small lattice fringes, indicating the presence of some incomplete crystalline SiO_2_ structures. Furthermore, the study investigated the effect of surface modification on the stability and thermal conductivity of water-based SiO_2_-coated graphene nanofluids. The findings demonstrated that SiO_2_ coating significantly enhanced the hydrophilicity of graphene, leading to improved dispersion stability and thermal conductivity of the nanofluid. It was also highlighted that SiO_2_ coating provides superior thermal conductivity enhancement to surfactant-assisted methods for preparing water-based graphene nanofluids.

**Fig. 19:**
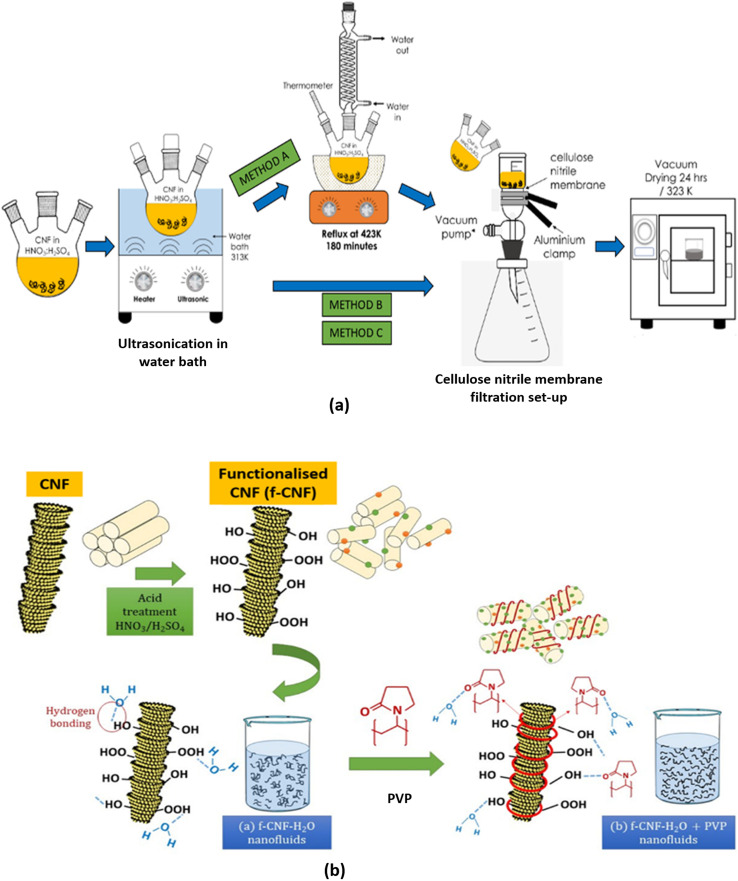
(a) Three different methods introduced to produce the surface-oxidised CNF, and (b) the mechanism of surface oxidation and PVP on the dispersion of CNFs in water-based liquids. Reproduced from ref. 75. Creative Commons Attribution 4.0 International License.

Carbon nanofibers (CNFs) are another class of nanomaterials with excellent heat transfer properties. However, their practical application is significantly limited due to poor dispersibility in most solvents. This instability is primarily attributed to their highly nonpolar surface, which prevents effective interaction and bonding with various matrix materials. To address this challenge, functionalization techniques—such as adding new functional groups or incorporating other materials such as metals or polymers—can convert hydrophobic CNFs into hydrophilic CNFs, thereby enhancing their dispersibility and stability in aqueous media.^9^ Two primary approaches are currently used to disperse carbon nanofibers: mechanical (or physical) methods and chemical methods. Mechanical methods, such as ultrasonication and high-shear mixing, physically separate nanotubes from each other. However, these techniques can also fragment long nanofibers, reducing their aspect ratio during processing, making them time-consuming and inefficient. Alternatively, chemical methods utilize surfactants or chemical moieties to modify the surface energy of nanotubes, improving their wettability, adhesion properties, and dispersion stability in solvents. For instance, Mohd Saidi *et al.*^75^ introduced surface oxygen functional groups *via* acid treatment on CNFs. The surface-oxidized CNFs were synthesized using three preliminary acid treatment methods: method A (CNF-MA), method B (CNF-MB), and method C (CNF-MC), each modifying commercial CNFs differently. In method A, a reflux treatment was included, whereas in methods B and C, reflux was excluded. Additionally, the ultrasonication duration for methods B and C was fixed at 2 hours and 6 hours, respectively, as illustrated in Fig. 19(a). Following oxidation, CNF-based nanofluids were prepared with concentrations ranging from 0.1 to 1.0 wt%, incorporating a fixed 10 wt% of polyvinylpyrrolidone (PVP). The mechanism of surface oxidation and the role of PVP in dispersing CNFs in water-based liquids are depicted in Fig. 19(b). Among the tested methods, method B was identified as the optimal approach for synthesizing surface-oxidized CNFs for nanofluid production. This selection was based on characterization results from Raman spectroscopy (higher ID/IG ratio), thermogravimetric analysis (TGA), and Fourier-transform infrared (FTIR) spectroscopy, indicating improved functionalization and dispersion stability.

Thus, it is evident that the surface oxidation of CNFs through acid treatment significantly enhances the thermal performance and stability of nanofluids by introducing oxygen functional groups such as hydroxyl (–OH) and carboxyl (–COOH) groups. The thermal conductivity of the pure base fluid at temperatures of 6 °C, 25 °C, and 40 °C was 0.546, 0.570, and 0.595 W m^−1^ K^−1^, respectively. However, the highest thermal conductivity of the surface-oxidized CNF-based nanofluid with PVP was recorded at 0.9 wt% concentration, reaching 0.647 W m^−1^ K^−1^ at 6 °C. At 0.7 wt% concentration, the nanofluid exhibited the highest thermal conductivity values of 0.666 W m^−1^ K^−1^ and 0.713 W m^−1^ K^−1^ at 25 °C and 40 °C, respectively. In other words, the thermal performance of the surface-oxidized CNF-based nanofluid with PVP showed an enhancement of 18.50% at 6 °C, while improvements of 16.84% and 19.83% were recorded at 25 °C and 40 °C, respectively. Notably, all the three highest enhancement percentages were achieved at a CNF concentration of 0.7 wt%, indicating that this concentration is optimal for maximizing the thermal conductivity improvement of the surface-oxidized CNF-based nanofluid with PVP. This improvement is attributed to the presence of oxygen functional groups on the CNF surface, which enhance interactions between water molecules and carbon particles, thereby improving the CNF hydrophilicity and preventing sedimentation in ultrapure water. Additionally, the pyrrolidone group in PVP facilitates homogeneous dispersion by reducing the boundary layer of suspended nanoparticles, preventing aggregation over extended periods and ultimately improving energy transfer efficiency.

### 6.2 Physical modification

#### 6.2.1 Optimization of the volume fraction of hybrid nanocomposites

**Fig. 20:**
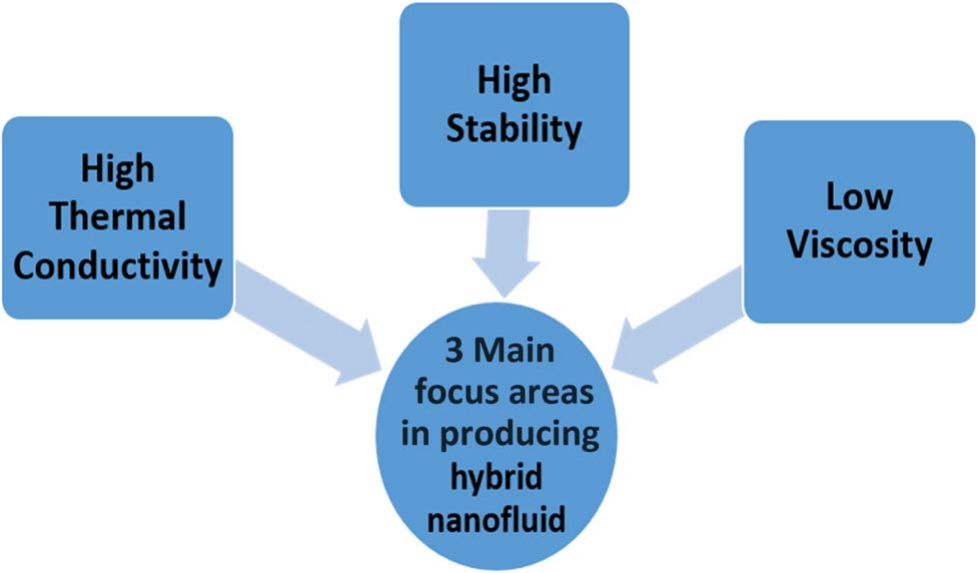
Three main focus areas to obtain water-based hybrid nanofluids: high thermal conductivity, stability and low viscosity.

One of the most debated topics in nanofluid research is the effect of volume fraction on the stability and thermal conductivity of the hybrid nanofluid. The volume fraction of nanoparticles significantly influences the stability, thermal conductivity and viscosity of hybrid nanofluids. Moreover, it was experimentally noticed that the volume concentration was directly proportional to the increase in thermal conductivity.^76,77^ Besides, a majority of previous papers have concluded that there is an urgent need to identify the optimum concentration of nanoparticles, which results in minimum viscosity, maximum thermal conductivity and high stability, as shown in Fig. 20.^78^

Furthermore, Suresh *et al.*^33^ demonstrated that the hybrid nanofluid with a high volume fraction of nanoparticles has less stability, and based on the study, it was suggested that adding a small percentage of hybrid nanoparticles to the cavity significantly improved stability and heat transfer properties. Additionally, they observed an increase in zeta potential from +30.1 mV to +46 mV as the volume concentration decreased from 2% to 0.1%. This indicates that the stability of hybrid nanofluid is dependent on the volume concentration of the nanoparticles, and the stability of the nanofluids at a higher concentration is poor. However, the thermal conductivity of Al_2_O_3_–Cu/water hybrid nanofluids with sodium lauryl sulphate (SLS) as a dispersant using an ultrasonic vibrator increased almost linearly with the particle volume fraction. This linear relationship between the measured thermal conductivity ratio and the volume concentration may be because of the formation of larger particle-free regions in the liquid that offer greater thermal resistances by the agglomerated particles. Compared to the base fluid (water), the Al_2_O_3_–Cu/water hybrid nanofluid showed improvements in thermal conductivity of 1.47%, 3.27%, 6.22%, 7.53%, and 12.11% for volume concentrations of 0.1%, 0.33%, 0.75%, 1%, and 2%, respectively. The experimental measurement of thermal conductivity showed a maximum enhancement of 12.11% for a volume concentration of 2%. Still in this study, there is no optimum value of volume fraction suggested with the enhancement of stability and thermal conductivity. The only conclusion based on this study is that when the volume fraction increases from 0.1% to 2%, the thermal conductivity increases, whereas stability improves when the volume fraction decreases from 2% to 0.1%. However, the thermal conductivity and viscosity of the hybrid increase with the increase in volume fraction, but the increase is higher in terms of viscosity, which is a challenge.

Similarly, the study conducted by Hemmat Esfe *et al.*^56^ examined the impact of nanoparticle volume fraction on the thermal conductivity and viscosity of Ag/MgO water-based hybrid nanofluids, in which the MgO particles measured 40 nm and the Ag particles 25 nm. The particle ratio of Ag to MgO was kept constant at 50 : 50, with volume fractions ranging from 0% to 2%. Surfactant CTAB was used in combination with ultrasonic vibration for 3 hours to maintain stability and address agglomeration issues. It is clear that the increase in nanoparticle volume fraction can increase the thermal conductivity; however, at very high concentrations, it can lead to a significant increase in viscosity. Moreover, as the volume fraction of nanoparticles increase, the distance between particles (free path) decreases. This increased contact between particles increases the frequency of lattice vibrations, further improving thermal conductivity with a higher temperature and volume fraction.^79^ While dynamic viscosity increases with volume concentration, it decreases with the increase in temperature. The study found that increasing the nanoparticle volume fraction led to higher thermal conductivity and dynamic viscosity of the nanofluid, but there is no focus given to the effect of volume fraction on the stability.

Similarly, Mousavi *et al.*^80^ examined the effect of nanoparticle volume fraction and temperature on the thermophysical properties of CuO/MgO/TiO_2_ ternary hybrid nanofluids. In this case, SDS was used as a surfactant, along with ultrasonic homogenization and magnetic stirring, to achieve homogeneous suspensions. The results showed that both dynamic viscosity and thermal conductivity of the ternary hybrid nanofluid increased with higher solid particle volume fraction and temperature. Notably, lower nanoparticle concentrations (0.1% to 0.3%) exhibited better stability and thermal conductivity compared to higher concentrations (0.4% to 0.5%), at which particles tended to aggregate. The highest thermal conductivity observed was 78.6% at a 0.1% volume fraction. Besides, Adogbeji *et al.*^47^ also proved that producing a hybrid nanofluid with low volumetric fraction can have better heat transfer properties. Therefore, in this study, a low volume fraction ranging from 0.00625% to 0.3% vol was used to prepare magnetic hybrid nanofluids (Fe_3_O_4_/TiO_2_). The result indicated that the hybrid nanofluid had a low volumetric fraction, while 0.00625 vol%, 0.0125 vol%, and 0.025 vol% demonstrated better stability with sedimentation factors (SF) of 8.89%, 9.82%, and 10.24%, respectively, compared to a higher volumetric fraction. Therefore, it is clear that a hybrid nanofluid with a low volumetric fraction also has capabilities to show better heat transfer with low pressure losses.

Zeng *et al.*^81^ also studied the effect of the volume fraction of graphene oxide/aluminum oxide nanoparticles (0.1, 0.25, 0.5, 0.75, and 1%) and temperature (25–50 °C) on the thermal conductivity. The results indicated that increasing the temperature from 25 °C to 50 °C and the volume fraction from 0.1% to 1% increased the thermal conductivity of the nanofluid up to 33.9%, with the former having a greater effect. Based on the results, compared to the base fluid, the largest improvement in thermal conductivity (33.9%) was achieved at a volume fraction of 1% and a temperature of 50 °C. As a result, this study also proved that the increase in temperature with volume fraction can improve the thermal conductivity and, in this study, there is less focus on the effect of the volume fraction to the stability of the hybrid nanofluid. Besides, Borode *et al.*^82^ have recently investigated the effect of volume fraction and temperature on the stability and thermophysical properties of the graphene/Fe_2_O_3_-based hybrid nanofluid. Based on the study, it was concluded that the nanofluid's viscosity increases with the addition of the hybrid nanoparticles and decreases as the temperature increases. Furthermore, the study shows that the thermal conductivity of the nanofluid is enhanced with increased addition of hybrid nanoparticles in the base fluid. However, there is still a lack of information on the effect of stability on the addition of the nanoparticles.

There is some type of argument from the researchers regarding the volume fraction of the nanoparticles in the nanofluid application: (1) higher nanoparticle concentrations lead to higher thermal conductivity. However, beyond a certain optimal threshold, nanoparticle aggregation becomes significant and impedes heat transfer. (2) Some researchers have observed that the enhancement in thermal conductivity with increased nanoparticle volume fractions is more pronounced at higher temperatures than at lower temperatures, due to the influence of temperature on the Brownian motion of nanoparticles.^54,83^ (3) Nevertheless, increasing the volume fraction of nanoparticles can also lead to deposition and agglomeration, which can reduce thermal conductivity and increase the viscosity of the nanofluid, creating additional challenges. (4) While thermal conductivity improves with both temperature and volume fraction, the effect of temperature is less pronounced at low volume fractions but becomes more significant at higher volume fractions. An excessive increase in the proportion of nanomaterials can lead to greater particle aggregation, reducing the surface-to-volume ratio and resulting in decreased thermal conductivity. However, there is still no clear statement on the optimum volume fraction of nanoparticles for better stability and also high thermal conductivity of hybrid nanofluids.

#### 6.2.2 Selection of suitable size, type and shape of nanoparticles

Another most debated topic in nanofluid research is the effect of nanoparticle size on the effective thermal conductivity of the fluids. Previous studies have shown conflicting results: some found a direct relationship between the nanoparticle size and effective thermal conductivity,^84^ while others reported an inverse relationship. Additionally, a few researchers have claimed that changes in nanoparticle size have no effect on the effective thermal conductivity of nanofluids at all. Initially, micrometer-sized particles were introduced into base fluids. However, these larger particles led to issues such as sedimentation, increased pressure drop, and erosion. As a result, nanoparticles with sizes less than 50 nm, including metallic, non-metallic, and polymeric particles, were introduced into heat transfer fluids. This innovation led to the development of a new class of heat transfer fluids known as nanofluids. Furthermore, Ambreen and Kim have conducted a systematic comparison of the effects of particle size on the thermal conductivity of nanofluids.^85^ Research has consistently shown that reducing the particle size below 100 nm enhances heat conductivity.^29^ This effect is more pronounced at higher fluid temperatures and particle densities.

As the particle size decreases, the increased random motion of nanoparticles promotes convection-like processes, leading to improved nanofluid conductivity. Smaller nanoparticles are more effective at enhancing conductivity.^34^ Nanoparticles can come in various shapes, including spherical, triangular, cubic, hexagonal, oval, prism, platelet, rod, tube, or spiral. Studies indicate that nanoparticles with a higher surface-to-volume ratio exhibit better thermal conductivity due to more efficient interphase heat exchange. For instance, nanotube-based nanofluids demonstrate high thermal conductivity. Nanoparticles with higher thermal conductivity contribute to the overall thermal conductivity of the nanofluid. For example, recent studies reveal that graphene has a very high thermal conductivity, so it is obvious that graphene nanofluid would show a higher thermal conductivity enhancement compared to other nanoparticles.^86^ Hybrid nanofluids containing nanoscale particles show higher thermal conductivity as the temperature and particle volume fraction increase, compared to those with micron-sized particles.

Besides the nanoparticle size, the shape of the nanoparticles influences the properties of the hybrid nanofluid. Hence, Ghadikolaei *et al.*^87^ studied the effect of shape and thermal characteristics of titanium oxide and copper nanoparticles on the final properties of water-based hybrid nanofluids. In addition, Iqbal *et al.*^88^ addressed the nanoparticle shape effect and novel features of hybrid nanofluid curvilinear transport (SiO_2_–MoS_2_/H_2_O). Based on the numerical investigation, it has been proven that the hybrid nanofluid has a rapid flow compared to the nanofluid; blade-shaped nanoparticles of the hybrid nanofluid have a maximum temperature, while the lowest temperature is observed for brick-shaped nanoparticles of nanofluids. Carbon nanoparticles, such as MWCNTs, are hydrophobic and possess high surface energy. MWCNTs, with their tubular shape, typically have a specific surface area greater than 200 m^2^ g^−1^, whereas nanoparticles like CuO have a specific surface area of around 50 m^2^ g^−1^. This high surface area contributes to the instability of carbon-based nanoparticles in polar base fluids, leading to clumping.

Another interesting impact of the nanoparticles was investigated by Xie *et al.*^89^ They mentioned that there is a relationship between the thermal conductivity and also specific surface area (SSA) of the nanoparticle suspensions. In this study, alumina particles were used with specific surface areas in the range of 5–124 m^2^ g^−1^. It is seen that the thermal conductivity first increases and then decreases with the increase in SSA, with the largest thermal conductivity at a particle SSA of 25 m^2^ g^−1^. We ascribe the thermal conductivity change behavior to twofold factors. First, as the particle size decreases, the SSA of the particle increases proportionally. Heat transfer between the particle and the fluid occurs at the particle–fluid interface. Therefore, a dramatic enhancement in thermal conductivity is expected because a reduction in particle size can result in large interfacial area. Second, the mean free path in polycrystalline Al_2_O_3_ is estimated to be around 35 nm, which is comparable to the size of the particle that was used. The intrinsic thermal conductivity of nanosized Al_2_O_3_ particles may be reduced compared to that of bulk Al_2_O_3_ due to the scattering of the primary carriers of energy (phonon) at the particle boundary. It is expected that the suspension's thermal conductivity is reduced with the increase in the SSA. Therefore, for a suspension containing NPs at a particle size much different from the mean free path, the thermal conductivity increases when the particle size decreases because the first factor is dominant. However, when the size of the dispersed NPs is close to or smaller than the mean free path, the second factor will govern the mechanism of the thermal conductivity behaviour of the suspension.

**Fig. 21:**
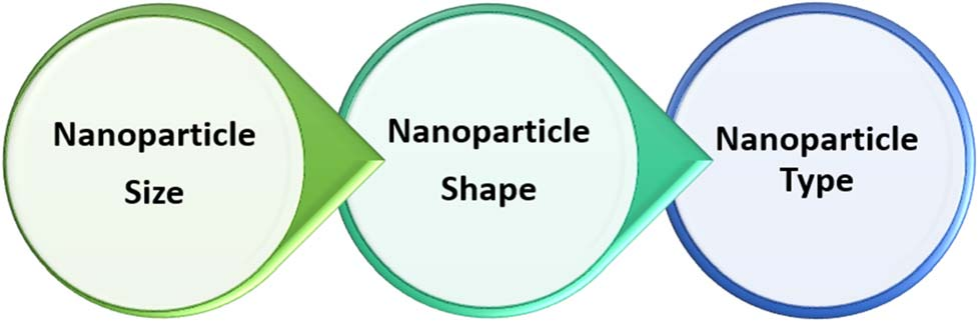
Factors that can affect the thermal stability and conductivity of the hybrid nanofluid.

Beside the nanoparticle shape, the type of nanoparticles can affect thermal conductivity and stability. Metal oxide nanoparticles such as TiO_2_, Fe_3_O_4_, ZnO, and Al_2_O_3_ offer an economical alternative to carbon-based nanoparticles. Among these, ZnO single nanofluids are particularly cost-effective, with very good optical properties and enhanced bandgap energy along with their environmental friendliness and stability nature.^83^ From both the economic and performance perspective, hybrid nanofluids often outperform single nanofluids. Al_2_O_3_, TiO_2_, and CuO nanoparticles are widely used across various applications due to their low cost, chemical stability, and effective heat transmission properties. Cu nanoparticles, known for their strong thermal conductivity, also represent a viable option. The combination of these nanoparticles may provide an effective solution for hybrid nanofluids. Tiwari *et al.*^90^ investigated the use of various metal oxides, such as CuO, MgO, and SnO_2_, combined with multi-walled carbon nanotubes (MWCNTs) in a weight ratio of 80/20. In this study, a CTAB surfactant was added at a ratio of 3/2 to stabilize the nanofluid. While alumina (Al_2_O_3_) and other ceramic materials are known for their excellent stability and chemical inertness, they have relatively low thermal conductivity compared to metallic nanoparticles. In contrast, metallic nanoparticles typically exhibit high thermal conductivity, but face challenges related to stability and reactivity. In conclusion, it is clear that the size and type of nanoparticles can influence the thermal conductivity, as shown in Fig. 21.

#### 6.2.3 Optimum ultrasonication time

Ultrasonication is a commonly used method to enhance the stability of hybrid nanofluids and disperse nanoparticles in a base fluid. Research has demonstrated that ultrasonication effectively breaks down nanoparticle agglomerations, thereby improving the stability of hybrid nanofluids. This is because the ultrasonic waves disrupt the van der Waals forces between nanoparticles, leading to increased dispersion within the medium. In other words, the increase in sonication time allows nanoparticles to be appropriately suspended in the base fluid, breaking up clusters and preventing agglomeration, resulting in a more stable and uniformly suspended network. This uniform suspension enhances the thermal conductivity value due to higher colloidal stability. It uses sound waves to break up particle agglomeration, allowing for homogeneous mixing in the solution.^41^ The duration and type of sonication have a significant impact on the stability of hybrid nanofluids. There are two types of ultrasonication: (1) direct sonication (probe sonication), in which the indirect sonication (ultrasonic bath) is immersed in the colloidal mixture and (2) indirect sonication, where the ultrasonic waves are transmitted to submerged nanofluid samples through the liquid.^91^ Among these two techniques, the probe sonicator provides superior outcome in terms of breaking the particle cluster and lowering the average cluster size.^20^

Some studies stated that, extending the sonication period enhances the stability of the hybrid nanofluid and this also will affect the viscosity of the hybrid nanofluid by breaking down bigger particles, lowering flow resistance, and reduce the viscosity.^41^ However, excessive sonication might cause particle re-aggregation can result in lower relative thermal conductivity across various solid volume fractions.^60^ As a result, selecting the correct sonication period is critical for obtaining increased stability and thermal conductivity. There is debate over the optimal sonication duration, as prolonged exposure can potentially induce defects in nanoparticles due to high-energy sonication fluids. For example, Sajid *et al.*^91^ studied the effect of sonication time of CuO, Fe_3_O_4_ and CNT water nanofluids using the two-step method with different ultrasonication times such as 30 min, 60 min, and 90 min for spectrum selective applications. The most stable water-based nanofluid was the CNT nanofluid with 0.004% concentration, and 90 min of ultrasonication resulted in an average of 67.6% and 74.6% higher absorbance than the stable CuO and Fe_3_O_4_ nanofluids, respectively.

**Fig. 22:**
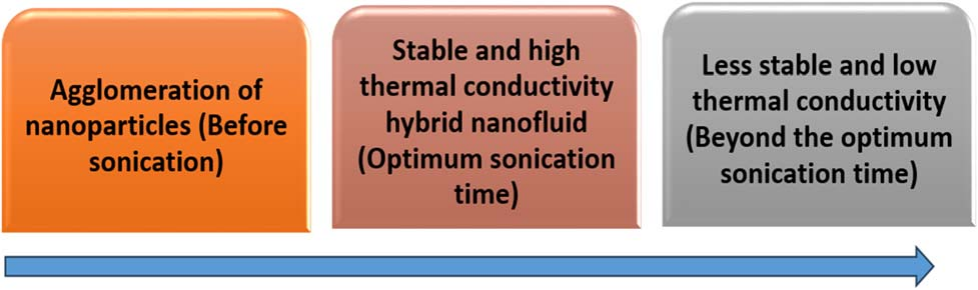
Effect of sonication time on the hybrid nanofluid.

Similarly, Asadi *et al.*^92^ studied the effects of ultrasonication time on the stability and thermal conductivity of MWCNT water-based nanofluids. It was found that extending the ultrasonication time until 60 min results in enhanced stability of the samples at all the nanoparticle concentrations, while prolonged ultrasonication deteriorates the stability; furthermore, extending the ultrasonication time leads to a gentle enhancement in thermal conductivity. The maximum conductivity was achieved by applying 60 min ultrasonication. Thus, it is concluded that 60 min ultrasonication is the optimum time in which the thermal conductivity and stability reached their highest point. However, when the sonication time exceeds the optimal duration, the thermal conductivity value decreases due to the destabilization of the nanosuspension.^93,94^ In conclusion, it is clear that the hybrid nanofluid exhibits stability and high thermal conductivity at the optimum sonication time, below which agglomeration occurs and beyond which low stability and thermal conductivity are observed, as shown in Fig. 22.

#### 6.2.4 pH variation

**Fig. 23:**
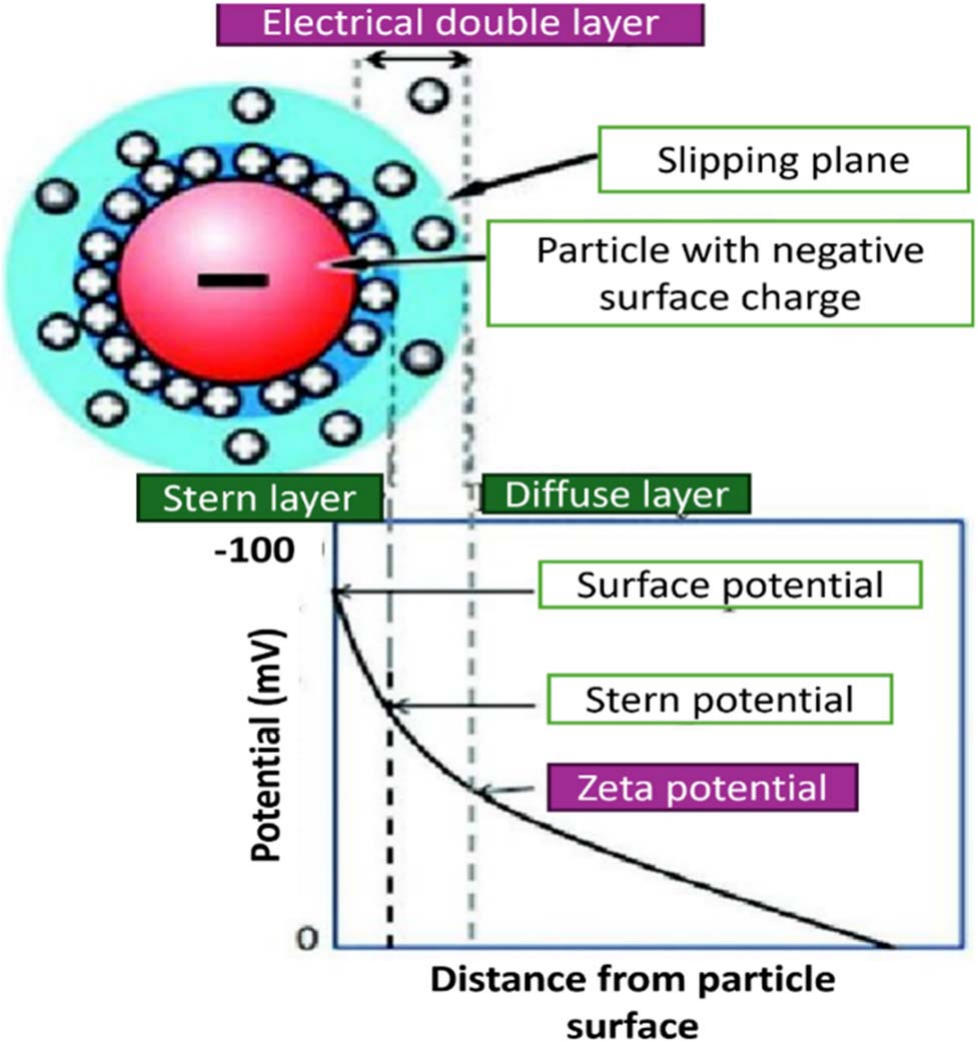
Zeta potential and the isoelectric point. Reproduced with permission.^95^ Copyright Wiley 2014.

The pH value is a key factor affecting surface electrical forces, particularly at the isoelectric point (IEP), where nanoparticles have no net electrical charge.^17^ IEP is the value of pH at which a particular molecule carries no net electric charge, or hydration forces are negligible. When the pH of nanofluids is equal or close to the IEP, nanofluids become unstable. The zeta potential is zero at the isoelectric point, and repulsive forces between nanoparticles suspended in the base fluid are zero and there is a tendency of coagulation.^17^ Adjusting the pH of a colloidal solution alters the electrical charge density on particle surfaces, which can either enhance or diminish repulsive interactions between suspended particles. In Fig. 23, the water layer surrounding particles is divided into two distinct regions: (1) the inner layer, known as the stern layer, and the (2) outer layer, or diffuse region.^96^ Ions are loosely bound in the diffuse layer and tightly bound in the stern layer. Together, the stern layer, diffuse layer, and surface charge form the electrical double layer (EDL), which is electrically neutral and contains both positively and negatively charged ions.^37^ The boundary within the diffuse layer where the particles and ions interact is called the zeta potential.^96^

Zeta potential refers to the potential difference between the dispersion medium and the stationary layer of fluid surrounding a dispersed particle. It is a crucial parameter for assessing the stability of colloidal suspensions, as it is highly sensitive to the pH of the solution. Altering the pH changes the electrical charge density on particle surfaces, which affects the strength of the repulsive forces between particles. IEP is where the positive and negative charges are balanced, resulting in minimal electrostatic repulsion between particles. Near the IEP, particles tend to agglomerate and reach their maximum size. Conversely, at pH values far from the IEP, the particle aggregation is minimal, and clustering is nearly absent.^97^ The magnitude of the zeta potential is a critical indicator of suspension stability. High zeta potential values, whether positive or negative, lead to repulsion between particles and prevent them from clumping together. Low zeta potential values, however, indicate weak repulsive forces and can lead to particle aggregation. Stable nanofluids must have a pH around 7, because deviations from this pH can lead to corrosion of the heat transfer surfaces, especially at elevated temperatures.^98^

Khairul *et al.*,^96^ investigated the effects of weight concentrations of nanoparticles as well as SDBS on the pH, zeta potential, particle size distribution, viscosity and thermal conductivity of alumina/CuO water-based nanofluids. These studies reported that increasing the volume fraction of nanoparticles and the weight concentration of surfactants led to a more alkaline pH. The zeta potential, which is highly dependent on the pH of the hybrid nanofluid, was evaluated for Al_2_O_3_/water and CuO/water nanofluids without any surfactant. The zeta potentials were around +30 to +40 mV for Al_2_O_3_/water and +14 to +28 mV for CuO/water, indicating positively charged nanoparticles. To modify the surface charges of the nanoparticles, negatively charged SDBS was added. SDBS dissociates in a solution to form phenyl sulfonic groups, which adsorb onto the nanoparticle surfaces, progressively increasing the surface's net negative charge and enhancing repulsive forces. The study observed that both nanofluids reacted similarly to changes in pH. The surface charge increased due to the more regular attack of potential-determining ions (H^+^, OH^−^ and phenyl sulfonic group). This may lead to an enhancement in the electrostatic repulsion force between nanoparticles, with the nanofluids showing notably decreased agglomeration and improved mobility, eventually boosting the heat transport method. After adding surfactants, the zeta potential values for Al_2_O_3_/water at 0.10 wt% SDBS and CuO/water at 0.15 wt% SDBS were −72.2 mV and −85.1 mV, respectively. Based on these studies, it is clear that controlling and optimising the surfactant concentration and pH can result in good corresponding relationship with the increment in the thermal conductivity of nanofluid. This is because a higher electrostatic force results in more free particles by enhancing the particle-to-particle distance, so that the distance exceeds the hydrogen bonding range between particles and further decreases the feasibility of particle coagulation and increases the dynamism of nanoparticles.

In conclusion, it is clear that the thermal conductivity and stability of the nanofluids were found to be influenced by pH. Shifting the pH away from the isoelectric point (IEP) increased the particle charge and repulsion, leading to more stable nanofluids and improved thermal conductivity. Proper surface charging of nanoparticles thus results in stable nanofluids with enhanced thermo-physical properties. A higher pH increases the negative charge on the particle surfaces and enhances adsorption.^52^

## 7 Relationship between the stability and thermal conductivity of water-based hybrid nanofluids with different types of nanoparticles

### 7.1 Water-based hybrid nanofluids

In this section, the recent available water-based hybrid nanofluids with different combinations of nanoparticles such as metals, metal oxides, carbon based and nanocellulose are discussed. This is because, suitable nanoparticle selection is an important task to achieve the necessary synergistic effect in hybrid nanofluids, and different combinations of nanoparticle ratios, types and concentrations can influence the performance of the hybrid nanofluid, such as stability and thermal conductivity. Moreover, in this section, steps or ways that previous studies have undertaken to increase the stability and thermal conductivity and the relationship between stability and thermal conductivity are explored.

#### 7.1.1 Combination of different types of metal and metal oxide-based hybrid nanofluids

The first type of nanoparticle combination in water-based hybrid nanofluids consists of metals with metal oxides, or metal oxides with metal oxides.^99^ For example, Moldoveanu *et al.*^100^ developed a water-based hybrid nanofluid by combining two different types of nanoparticles: Al_2_O_3_ and SiO_2_. The nanoparticle suspensions were subjected to ultrasonic vibration for approximately 60 minutes to achieve a uniform dispersion. The study revealed that the thermal conductivity of the nanofluids increased with the volume fraction of both Al_2_O_3_ and SiO_2_ nanoparticles, as well as with the increase in temperature. Additionally, SiO_2_ nanoparticles exhibited a higher thermal conductivity than Al_2_O_3_ due to their inherently superior thermal conductivity. Maximum enhancement in the thermal conductivity of 17.96–23.61% was achieved for a maximum volume fraction of 0.5% Al_2_O_3_ + 2.5% SiO_2_ and a temperature range of 20–50 °C. One can clearly notice that the thermal conductivity is increasing linearly with temperature, as well as the overall volume fraction of hybrid nanofluids. This enhancement in thermal conductivity with the increase in temperature is attributed to the Brownian motion and enhanced interactions between nanoparticles. However, this study did not focus on the stability of the hybrid nanofluids, but this study gives a clear statement that the volume fraction of nanoparticles and temperature influence the thermal conductivity variation.

**Fig. 24:**
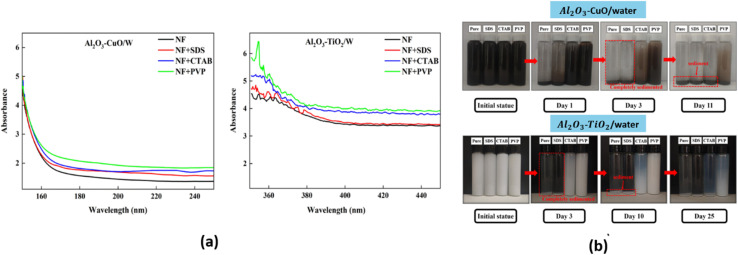
(a) UV-vis result and (b) visual sedimentation result for Al_2_O_3_–CuO/water and Al_2_O_3_–TiO_2_/water with different surfactants. Reproduced with permission.^101^ Copyright Elsevier 2021.

However, another study conducted by Ma *et al.*^101^ focuses on the effect of adding different types of surfactants such as SDS (anionic), CTAB (cationic), and PVP (non-ionic) on the stability of Al_2_O_3_/CuO and Al_2_O_3_/TiO_2_ water-based hybrid nanofluids at different mass concentrations of surfactants (0.005 wt% to 0.05 wt%). The volumetric fraction of the hybrid nanofluid is fixed to 0.005 vol%, nanoparticle ratios (Al_2_O_3_ : TiO_2_ and Al_2_O_3_ : CuO) are set to 20/80, while the temperature varies within the range of 20–60 °C. Moreover, ultrasonication vibration and magnetic stirring are for 60 min and 20 min, respectively, to guarantee the stability of the prepared nanofluids. The results indicated that PVP with 0.005 wt% was the most effective surfactant for stabilizing the mentioned hybrid nanofluids based on the UV-vis spectrophotometry, TEM images and visual observation (sedimentation method) results. The higher the UV-vis absorbance values, the better the dispersion of the nanofluids. The absorbance of nanofluids with PVP surfactants increased by approximately 32.7% for Al_2_O_3_/CuO nanofluids and 15.8% for Al_2_O_3_/TiO_2_ nanofluids compared to those without surfactants, as shown in Fig. 24(a). Furthermore, the TEM images indicate that hybrid nanofluids with PVP surfactants exhibit the most uniform nanoparticle dispersion. Visual observations also confirm minimal sedimentation for up to 25 days, with PVP performing the best, followed by CTAB and SDS, as shown in Fig. 24(b). This indicates that only 0.005 wt% of PVP surfactant is needed to produce a highly stable hybrid nanofluid, offering a cost-effective and efficient way to enhance the thermophysical properties and stability of the hybrid nanofluid. Therefore, the optimum concentrations of the PVP surfactant are 0.005 wt% and 0.01 wt% for Al_2_O_3_/CuO and Al_2_O_3_/TiO_2_ hybrid nanofluids, respectively. These values show the highest thermal conductivity increment for the studied temperature. Based on these studies, it was clear that it is crucial to know the optimal level of surfactant, known as the critical micelle concentration (CMC), as exceeding this level can decrease the stability and thermal conductivity of the hybrid nanofluid. Excessive surfactant can lead to an increased number of micelles, causing surfactant molecules and nanoparticles to aggregate into larger clusters. This aggregation diminishes Brownian motion and micro-convection, resulting in a decline in thermal conductivity enhancement.^101^

Moreover, Mousavi *et al.*^102^ investigated the physicochemical properties of a tertiary hybrid water-based nanofluid consisting of CuO/SiO_2_/CaCO_3_, positing that tertiary hybrid nanofluids exhibit superior physicochemical properties to mono- or binary nanofluids. They emphasized that nanofluid stability is crucial for its long-term applicability in various fields. SiO_2_, renowned for its excellent thermal and hydrodynamic properties, is widely used in hybrid nanofluid production and attracts significant research interest across diverse industries. CaCO_3_ offers benefits such as low cost, eco-friendliness, and the ability to stabilize other nanoparticles in nanofluids by acting as a protective coating or stabilizer. Furthermore, CuO, known for its high thermal conductivity and Brownian motion with low viscosity, can enhance the overall energy efficiency of the system. However, these nanoparticles tend to agglomerate and sediment under various conditions. To mitigate this issue, Mousavi *et al.*^102^ incorporated SDS as a surfactant to improve the stability of the tertiary hybrid nanofluid and optimized the sonication time to achieve the most stable formulation. Their study identified the optimal nanoparticle volume ratio as 60/30/10, with a total volume fraction of 0.5% in water as the base fluid. SDS stabilizes nanofluids by supercharging the nanoparticles and improving their electrical repulsion. This phenomenon is caused by the absorption of SDS onto nanoparticle surfaces, altering their surface characteristics, increasing their specific surface area, and enhancing their adsorptive capacity. The ideal SDS concentration should be below the critical micelle concentration (CMC). The highest thermal conductivity achieved was a 78.6% improvement at a 0.5% total volume fraction.

While most studies on hybrid nanofluids focus on the effect of volume fraction on their thermophysical properties, V. Wanatasanapan *et al.*^103^ explored the impact of nanoparticle mixing ratios on the properties of hybrid nanofluids. In their study, they fixed the volume fraction of TiO_2_ and Al_2_O_3_ nanoparticles at 1.0% but varied the mixing ratios of TiO_2_ and Al_2_O_3_ using a two-step technique. The results indicated that the hybrid nanofluid with a TiO_2_/Al_2_O_3_ mixing ratio of 50/50 at 70 °C exhibited the highest thermal conductivity at 1.134 W mK^−1^, while the hybrid nanofluid with a TiO_2_/Al_2_O_3_ mixing ratio of 80/20 at 30 °C showed the lowest thermal conductivity, with PVP as a surfactant at a ratio of 1/10. These findings confirm that the thermal conductivity of the nanofluid improves steadily as the TiO_2_ mixing ratio increases, reaching its peak when the TiO_2_/Al_2_O_3_ mixing ratio is 50/50. Furthermore, the Brownian motion effect of the nanoparticles at high temperatures led to a notable rise in the hybrid nanofluid's thermal conductivity compared to water as the temperature increased. The created hybrid water-based nanofluid exhibited a linear trend of shear stress at all shear rates and temperatures, indicating Newtonian flow behavior at all nanoparticle mixing ratios and temperatures. Additionally, the hybrid nanofluid with different nanoparticle mixing ratios showed variations in dynamic viscosity at all temperatures. The hybrid nanofluid with a ratio of 50/50 achieved moderate stability, with a zeta potential value of 29.6 mV.

Next, Mousavi *et al.*^104^ studied the thermophysical characteristics of a MgO/TiO_2_ water-based hybrid nanofluid with the addition of the surfactant SDS and the aid of ultrasonic homogenizer and a magnetic stirrer. They noted that the addition of surfactants reduced the surface tension from 0.0713 to 0.0432 N m^−1^, and ultrasonication helped to distribute the nanoparticles more evenly. However, as the volume fraction of hybrid nanoparticles increased, the surface tension also increased due to the van der Waals forces between particles at the water/air interface, leading to enhanced surface free energy. This issue was mitigated by adding MgO nanoparticles or 80 wt% MgO and 20 wt% TiO_2_ hybrid nanoparticles, which could reduce the surface tension between air and water, providing a potential drag reduction technique. The thermal conductivity of 80 wt% MgO and 20 wt% TiO_2_ hybrid nanoparticles is improved by 21.8% at a temperature of 50 °C.

Furthermore, in a recent study conducted by Alklabi *et al.*,^13^ a Fe_3_O_4_–SiO_2_ water-based hybrid nanofluid was prepared for plate heat exchanger applications, and fewer studies used silica-coated magnetite. Black iron oxide (Fe_3_O_4_) has the highest magnetic property and silicon dioxide (SiO_2_) is characterized by its high hardness, chemical stability, toughness, and yield strength. The Fe_3_O_4_–SiO_2_ nanoparticles were synthesized by the method of chemical co-precipitation and *in situ* growth technique. Besides that, in order to increase the stability of the hybrid nanofluid, SDBS was used as a surfactant of 1/10th weight of the nanoparticles. Moreover, the Fe_3_O_4_ nanoparticles prepared by a chemical coprecipitation method were mixed in a nitric acid and sulfuric acid solution for 3 days in order to form a carboxyl group (COOH) on the Fe_3_O_4_ nanoparticles. Later, this carboxyl group supported the SiO_2_ nanoparticles during the *in situ* growth for the preparation of the Fe_3_O_4_–SiO_2_ nanoparticles. Next, after the preparation of nanofluids, the solution pH was measured and observed to be neutral. Based on the study, it was concluded that by using 1.0 vol% of Fe_3_O_4_–SiO_2_/water hybrid nanofluids, the thermal conductivity was enhanced by 27.24% at a temperature of 60 °C and the viscosity of 1.0% vol of nanofluid increased by 50.76% at a temperature of 20 °C against the water data. Moreover, the specific heat of Fe_3_O_4_–SiO_2_ hybrid nanofluids was lower than that of the mono Fe_3_O_4_/water and SiO_2_/water nanofluids, which is an advantage because under the fixed rate of heat flow, the hybrid nanofluids absorbs more heat than the mono nanofluids. However, the viscosity of Fe_3_O_4_–SiO_2_ hybrid nanofluids is higher than that of the mono nanofluids, which is a disadvantage as the pressure drop and friction factor become higher, which can lead to an increase in pumping power. However, there is less focus given to the evaluation of the stability of the hybrid nanofluid.

However, Taherialekouhi *et al.*^57^ studied the stability and thermal conductivity of water-based hybrid nanofluids of graphene oxide (GO)/Al_2_O_3_ as cooling hybrid nanofluids at a fix volume fraction of nanoparticles of 50%, temperature range of 25–50 °C and volume fractions of 0.1, 0.25, 0.5, 0.75, and 1%. It is a novel hybrid nanofluid as no previous study used such nanoparticles in producing hybrid nanofluids. This combination of nanoparticles can improve the thermal conductivity due to the presence of graphene nanostructures with high thermal conductivity with a lower total cost due to the lower price of alumina nanoparticles than graphene. As a result, the temperature increases from 25 °C to 50 °C, the volume fraction increases from 0.1% to 1% and the thermal conductivity of the nanofluid also increases. The maximum increase in thermal conductivity was up to 33.9% at a volume fraction of 1% and a temperature of 50 °C, due to a higher number of nanoparticles in the base fluid and the number of intermolecular collisions. The rise in temperature causes the molecular bonds to be weakened in the fluid layers and increases the nanoparticle movement, which causes more collision between nanoparticles, and finally the thermal conductivity of the nanofluid increases. In addition, when the fluid temperature rises, the particle dispersion would be higher, and the Brownian force becomes stronger, causing the thermophysical phenomenon to occur, so that the nanoparticles tend to disperse faster in warmer regions and slower in colder regions, as well as the movement from warm areas to cold regions, resulting in the dispersion of nanoparticles in all nanofluids and an increase in thermal conductivity. Besides that, the combination of such nanoparticles is cost effective and can reduce the viscosity, as the use of Al_2_O_3_ nanoparticles in the combined nanofluid improves the physical structure of GO nanoparticles, which in addition to reducing the viscosity of the nanofluid compared to the nanofluid of water/graphene oxide, reduces the cost of the produced nanofluid with improved properties. Lastly, the stability evaluation was conducted based on the size distribution of nanomaterials using the dynamic light scattering (DLS) method. Therefore, the hybrid nanofluid is considered stable, as there is no significant change in the size distribution of nanoparticles after the 30 days.

Apart from the combination of metal oxide nanoparticles, there are water-based hybrid nanofluids that combine metal oxide with metal nanoparticles. Aparna *et al.*^105^ produced a water-based hybrid nanofluid using Al_2_O_3_ and Ag nanoparticles with PVP as a surfactant. The addition of the surfactant was confirmed by the FTIR test, with a noticeable peak between 3200 and 3600 cm^−1^, attributed to the stretching vibration of O–H and N–H bonds present in PVP. The thermal conductivity of the hybrid nanofluid with a mixture ratio of 50/50 of Al_2_O_3_/Ag showed the greatest thermal conductivity compared to the 70/30 and 30/70 ratios and the Al_2_O_3_-based nanofluid. However, it had nearly the same thermal conductivity as the Ag-based nanofluid. Therefore, this hybrid nanofluid can be a more cost-effective substitute for aqueous Ag nanofluids in applications such as solar energy. Moreover, the stability of the hybrid nanofluid with a ratio of 50/50 showed the highest stability for 24 hours. The study also found that the sonication time had a direct influence on stability and thermal conductivity, with an optimal sonication period of 2 hours. Beyond 2 hours, the thermal conductivity decreased due to nanoparticle clustering, which reduced the effective surface area-to-volume ratio and, consequently, the effective transfer area of the particles, lowering the fluid's heat conductivity. Additionally, the thermal conductivity of the Al_2_O_3_/Ag nanoparticle water-based hybrid nanofluid increases with the nanoparticle loading. The temperature significantly impacts the average thermal conductivity at higher volume concentrations, while the change is negligible at lower volume concentrations. The primary causes of the temperature-dependent rise in thermal conductivity are the increased thermal energy and Brownian motion of the dispersed nanoparticles. As the volume concentration increases, so does the number of nanoparticles. Experimental results demonstrate that, for all types of nanofluids, at a constant temperature, thermal conductivity increased with the increase in the volume concentration of nanoparticle loading.

Furthermore, there is the production of water-based hybrid nanofluids combining metal and metal nanoparticles. For example, Nagendarmma *et al.* produced a hybrid nanofluid combining copper (Cu) and alumina alloy (AA7072) nanoparticles.^106^ However, there is limited research on metal–metal hybrid nanofluids and metal oxide-metal water-based hybrid nanofluids compared to metal oxide-metal oxide water-based hybrid nanofluids. This may be due to the properties of these nanoparticles. Metallic nanoparticles have greater thermal conductivity and reactive chemical properties but are more expensive than metal oxide nanoparticles, which have steady chemical inertness, are cheaper, and have lower thermal conductivity. Therefore, hybridizing such nanoparticles can result in hybrid nanofluids with superior heat transfer and flow behaviour.^107^ Moreover, in order to increase the stability and thermal conductivity, steps such as the addition of surfactants as well as monitoring volume fraction and temperature have been undertaken by researchers. Mostly the PVP and SDS or SDBS surfactant was used. Table 5 shows the summary of the different combinations of metal and metal oxide water-based hybrid nanofluids.

**Table 5:** Combination of metal and metal oxide water-based hybrid nanofluids

NPs 1	Stability improvement	Vol% & ratio of NPs	Stability & thermal conductivity (*T*_c_)	Ref.
**Metal oxide-metal oxide water-based hybrid nanofluid**				
Al_2_O_3_ : SiO_2_	Ultrasonic vibration (60 min)	Vol% of 0.5% Al_2_O_3_ + 2.5% SiO_2_	*T*_c_: 17.96–23.61%	100
Al_2_O_3_ : CuO	Surfactant (PVP) & ultrasonication vibration (60 min) &	0.005 wt% (Al_2_O_3_–CuO)	Stability up to 25 days	101
Al_2_O_3_ : TiO_2_	Magnetic stirrer (20 min)	0.01wt% (Al_2_O_3_–TiO_2_) (20 : 80)	*T*_c_: up to 12–14%	
CuO : CaCO_3_ : SiO_2_	SDS & ultrasonication	*φ*: 0.5 vol% (60 : 30 : 10)	Stability 8 days	102
			*T*_c_: up to 51.2%	
TiO_2_ : Al_2_O_3_	PVP	*φ*: 1.0 vol% (50 : 50)	Zeta: +29.6 mV	103
			*T*_c_: 1.134 W mK^−1^	
MgO : TiO_2_	SDS	*φ*: 0.3 vol% (80 : 20)	*T*_c_: 21.8%	104
Fe_3_O_4_ : SiO_2_	SDBS	*φ*: 1.0 vol%	*T*_c_: enhanced by 27.24%	13
Graphene oxide (GO) : Al_2_O_3_		*φ*: 1.0 vol% (50 : 50)	*T*_c_: improved 33.9%	57

**Metal–metal oxide water-based hybrid nanofluid**				
Al_2_O_3_ : Ag	PVP	*φ*: 0.1 vol% (50 : 50)	*T*_c_: 23.82%	105
			Stability: 24 h	

**Metal–metal water-based hybrid nanofluid**				
Cu : alumina alloy (AA7072)	—	—	—	106

#### 7.1.2 Combination of different types of carbon-based hybrid nanofluids with metal or metal oxide nanoparticles

Carbon-based nanoparticles (CNT) such as single-walled CNTs (SWCNTs), multi-walled CNTs (MWCNTs), and graphene (GE) are hydrophobic, leading to quick sedimentation due to strong van der Waals interactions, which limits their use in nanofluids. Despite this, their special structure, with a high surface-to-volume ratio and excellent thermal conductivity, draws significant interest for heat transfer applications.^108^ For instance, MWCNTs exhibit several superior characteristics including ultra-high thermal conductivity (over 3000 W m^−1^ K^−1^), chemical stability, specific surface area of over 200 m^2^ g^−1^ and excellent mechanical properties, making them better candidates for nanofluids than other types of nanoparticles. Graphene, which is a new form of carbon nanomaterials, shows an extremely high thermal conductivity of 5000 W mK^−1^.^109^ However, drawbacks such as poor stability and increased viscosity hinder the development of CNT nanofluids. Additionally, CNTs have an extremely high surface energy, which results in instability in polar base fluids, leading to clumping. To address this, researchers have synthesized CNT-based hybrid nanofluids by adding other types of nanoparticles such as metal oxides.

For example, Giwa *et al.*^48^ mixed functionalised MWCNTs with γ-Fe_2_O_3_ to produce a hybrid nanofluid at a mixing ratio of 80/20 with different temperatures (15 °C to 55 °C) and volume fractions (0.1–1.5%). This hybrid nanofluid demonstrated a maximum improvement of 35.7% in viscosity and 1676.4% in electrical conductivity compared to water. Moreover, the MWCNT/Fe_2_O_3_ water nanofluids were found to be stable and well suspended. In this study, SDS was used as a surfactant. An absorbance range of 3.0–3.8 with wavelengths of 287–264 nm was measured for the hybrid nanofluids at different volume concentrations (0.1–1.5%) using UV-visible spectrophotometry. It can be noticed that the absorbance increased with the increase in the volume concentration and the increased suspension of the nanoparticles into water altered the values of both the absorbance and the wavelength. Moreover, based on the sedimentation method, it was noticed that there was no sedimentation noticed after a month upon inspection. Moreover, Scott *et al.*^62^ synthesized an MWCNT/Al_2_O_3_ water-based hybrid nanofluid with different volume fractions (0.05%, 0.1%, 0.15%, and 0.2%) while maintaining a fixed nanoparticle ratio (MWCNT/Al_2_O_3_ at 90/10). Similarly, SDS was added to improve the stability and reduce the nanoparticle agglomeration. This hybrid nanofluid was designed for applications such as computer chips, solar collectors, and power electronics to prevent overheating. Stability tests using sedimentation techniques revealed no sedimentation or color changes even after two months. The study identified a volume concentration of 0.10% as optimal for natural convection heat transfer. Although the focus was primarily on stability and heat transfer capacity, thermal conductivity was also considered. In another study, MWCNTs were combined with ZnO at a mixing ratio at 50/50 wt% to produce a water-based hybrid nanofluid on the PV/T system's performance compared to both water-cooled and uncooled configurations.^110^ The nanofluid coolant in a photovoltaic thermal (PV/T) system achieved the greatest reduction in panel temperature, with a 14.9 °C decrease (33.7%) compared to uncooled panels and the incorporation of the hybrid nanofluid resulted in a peak thermal efficiency of 51.3%.

Furthermore, Tiwari *et al.*^45^ considered the 4S, which are synthesis, sonication, surfactant and stability, for the thermal stability of water-based hybrid nanofluids consisting of MWCNTs and CeO_2_ at a mixing ratio of 20/80 with varying ultrasonication times (30, 60, 90, 120, 150, and 180 min) and different types of surfactants including anionic (SDBS and SDS), cationic (CTAB and dimethylammonium chloride (DDC)) and polymer (PVP and GA), which was added to the base fluid with different nanoparticle-to-surfactant mixing ratios (5 : 0, 4 : 1, 3 : 2, 2 : 3 and 1 : 4). The results revealed that the nanofluid with a nanoparticle-to-surfactant ratio of 3/2 and CTAB as the surfactant achieved the highest zeta potential of 48 mV and remained stable for over 90 days with an optimal sonication time of 90 minutes. The study underscores that stabilization is closely linked to enhancement in thermal conductivity. The addition of surfactants was crucial for improving stability, with different surfactants yielding varying zeta potential values and stability durations. Specifically, CTAB, a cationic surfactant, and SDBS, an anionic surfactant, ensured stability for approximately 30 days and 90 days, respectively.^45^ This stability was attributed to the effective and homogeneous coating of nanoparticles by the surfactants, which created a uniform surfactant layer that provided excellent electrostatic repulsion against van der Waals forces, thus preventing agglomeration in the hybrid nanofluid suspension. In addition to surfactants, the duration of ultrasonication plays a crucial role in enhancing the stability of hybrid nanofluids.^45^ Increasing the sonication time improves the absolute zeta potential, which directly contributes to the stability of the hybrid nanofluid. However, sonication beyond 90 minutes at certain volumetric concentrations can lead to decreased stability and a lower absolute zeta potential value. Therefore, 90 minutes is generally considered the optimal sonication time. It has been observed that exceeding this optimal duration can adversely affect stability, reducing the colloidal stability of the suspension. Consequently, careful control and optimization of sonication time are essential for maintaining the stability and performance of hybrid nanofluids.

In addition to surfactant addition and optimization of ultrasonication time, researchers have explored functionalization methods, also known as surface modification, to enhance the stability of CNT-based hybrid nanofluids. These methods include both non-covalent and covalent functionalization, as well as mixing CNTs with metal or metal oxide nanoparticles, to improve the dispersion and thermal characteristics of the base fluids. Covalent functionalization involves forming chemical bonds with the CNTs, where the nanoparticles have at least one pair of electrons that create a covalent link. This process improves dispersion, processability, and reactivity of the nanoparticles. As a result, the thermal conductivity of CNT-based hybrid nanofluids increases with better dispersion. Due to their hydrophobic nature, CNTs typically have poor dispersion in water without modification or surfactants.

**Fig. 25:**
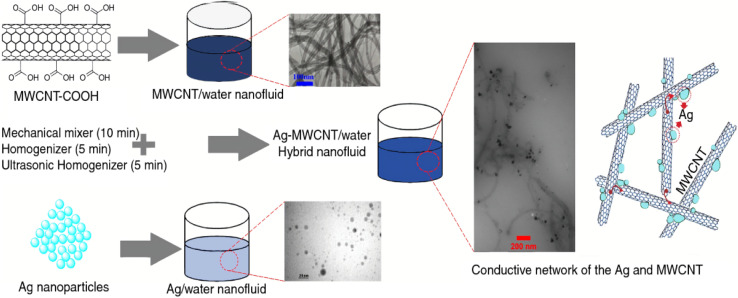
Decoration of Ag nanoparticles on the surfactant of the MWCNTs. Reproduced with permission.^111^ Copyright Springer Nature 2020.

Therefore, functionalization is employed to enhance the thermal conductivity of water-based hybrid nanofluids by combining MWCNTs with other nanoparticles. For example, functionalizing MWCNTs with COOH functional groups to alter their behaviour from hydrophobic to hydrophilic is believed to enhance the stability and thermal conductivity of the hybrid nanofluid.^111^ A study decorated COOH-functionalized MWCNTs with silver nanoparticles (Ag) to prepare a water-based hybrid nanofluid. Morphological tests confirmed that Ag nanoparticles were effectively decorated on the COOH-functionalized MWCNTs, showing a well-distributed presence on their surfaces, as shown in Fig. 25. This modification provides sites for Ag nanoparticles to bond with the functionalized MWCNTs. The study found that the improvement in thermal conductivity is linked to both temperatures rise and nanoparticle volume fraction. As the temperature increases, the kinetic energy of the nanoparticles increases, leading to enhanced thermal conductivity through increased Brownian motion. In other words, thermal conductivity increases nonlinearly with both volume fraction and temperature. The results indicate that both higher volume fractions and temperatures boost the thermal conductivity of the hybrid nanofluid. However, the temperature has a minimal impact on low concentrations but becomes more significant at higher concentrations. The modified MWCNTs with Ag demonstrated a 47.3% improvement in thermal conductivity at 50 °C.^111^ Nonetheless, the study did not address the stability of the hybrid nanofluid.

Besides that, Bakhtiari *et al.*^112^ proposed that thermal conductivity can be significantly enhanced by optimizing two key variables: temperature and nanoparticle concentration in the base heat transfer fluid. They developed a hybrid nanofluid with the combination of TiO_2_ and GE in a water-based solution at a temperature in the range of 25 to 75 °C and a nanoparticle concentration of 0.005 to 0.5% with the addition of carboxymethyl cellulose as a surfactant (CMC). TiO_2_ was added to lower the costs and improve the stability of GE/water nanofluids, leveraging its high surface-to-volume ratio to minimize particle clustering and achieve a more stable thermodynamic state. The results indicated that the enhancement of thermal conductivity with the increase in solid volume fraction is more pronounced at a higher temperature.^112^ However, the effect of increase in volume fraction on thermal conductivity was larger than the effect of temperature. The result indicates that thermal conductivity at a volume fraction of 0.5% and a temperature of 75 °C was increased by 27.84% compared to the base fluid. As the volume fraction increased, the number of particles also grew, leading to particle clustering. Under these conditions, heat transfer through the solid phase became more efficient than through the liquid phase, thereby enhancing the thermal conductivity. Additionally, elevated temperatures increased nanoparticle mobility, facilitating greater interactions between surface atoms and fluid molecules, which further improved the relative thermal conductivity. However, in this study, less attention is paid to the stability of the hybrid nanofluid.

Furthermore, Bhatti *et al.*^113^ developed hybrid nanofluids using a combination of silica (SiO_2_) and diamond (C) nanoparticles that are not commonly used in hybrid nanofluids for solar collectors, which require high thermal conductivity, large critical heat flux, high specific heat, and an enhanced heat transfer coefficient. The result was discussed based on the numerical outcomes. Next, Shaik *et al.*^114^ produced a hybrid water-based nanofluid by the incorporation of 2 different types of nanoparticles such as Cu/GE at a constant concentration of Cu of 0.04 vol% but different GE volume fractions from 0.01 to 0.1 vol%. This hybrid nanofluids display excellent stability against aggregation for up to 7 weeks, as proven by higher zeta potential values at 0.04 : 0.025 vol% of Cu/GE. The thermal conductivity of water-based hybrid nanofluids increases with the increase in the hybrid nanoparticle loading, due to the metallic nature of Cu, and the 2D structure of GE and CNT is affordable, having a high thermal conductivity even at low volume concentrations; however, SiO_2_ has low thermal conductivity but has really good stability with CNTs in water-based fluids. As a result, Dalkılıç *et al.*^115^ synthesised a CNT/SiO_2_ water-based hybrid nanofluid. However, sedimentation can be observed when the volume fraction of CNT-SiO_2_ is 2%. This issue was overcome by adding 10% GA, which shows better stability up to 120 h. In all samples, maximum enhancement of thermal conductivity was measured at 1% (80% CNT : 20% SiO_2_) and maximum enhancement was 26.29%. Most studies indicate that the nanoparticle volume concentrations of less than 1% are typically used to minimize unwanted increases in dynamic viscosity while maintaining optimal thermal conductivity. This approach aims to achieve high thermal conductivity while minimizing the impact on the dynamic viscosity of the nanofluid, ensuring effectiveness in fluid mechanics and heat transfer applications. Table 6 presents the summary of the different combinations of carbon-based and water-based hybrid nanofluids.

**Table 6:** Carbon-based and water-based hybrid nanofluids

NPs	Stability improvement	Vol%/ratio	Stability/thermal conductivity (*T*_c_)	Ref.
**MWCNT**				
Functionalised MWCNTs : γ-Fe_2_O_3_	SDS	(20 : 80)	No sedimentation even after a month	48
MWCNT : Al_2_O_3_	SDS	*φ*: 0.1vol% (90 : 10)	No sediment for 2 months	62
Acid functionalized MWCNT : ZnO	—	*φ*: 0.1 wt%	Greatest reduction in panel temperature	110
MWCNT : CeO_2_	CTAB sonication (90 min)	*φ*: 0.3 and 0.75 (20 : 80)	90 days	45
MWCNTs-COOH : Ag	Modification	(0.16 vol% MWCNT-COOH	*T*_c_: improved by 47.3% at 50 °C	111
		0.04 vol% Ag)		

**CNT**				
CNT : Cu		*φ*: 0.05% wt	Stability: 6 months	46
			*T*_c_: 8.82% (Cu/CNTs)	
CNT : Ag			*T*_c_: 11.94% (Ag/CNTs)	
CNT : SiO_2_	Surfactant (GA)	*φ*: 1% (80 : 20)	Stability *T*_c_: up to 120 h enhancement up to 26.29%	115

**Graphene**				
Graphene : TiO_2_	CMC (carboxymethyl cellulose salt)	*φ*: 0.5 vol%	*T*_c_: 27.84%	112
Graphene : Cu	—	Vol%: 0.025 : 0.04	Stability: up to 7 weeks (−65.5 mV)	114

**Diamond (C)**				
C : SiO_2_				113

### 7.2 Water/ethylene glycol-based hybrid nanofluid

Usually, the concentration of EG is lower than that of water due to EG's lower thermal conductivity. Therefore, researchers commonly use an EG/water ratio of 40/60. Although water is an affordable and efficient heat transfer fluid, it freezes below 0 °C. This issue can be resolved by adding EG to water, resulting in a high freezing point of −35 °C for the mixture. Additionally, the EG/water combination increases the boiling point, prevents corrosion, and improves the heat exchange efficiency.^116^

Furthermore, Ma *et al.*^51^ studied different mixtures of nanoparticles such as Al_2_O_3_/TiO_2_, Al_2_O_3_/CuO, and Al_2_O_3_–Cu in an EG–W-based fluid at a fixed volume fraction of 1.0 vol% at a temperature in the range from 20 to 60 °C. They emphasized that the nanoparticle mixing ratio is a crucial factor in determining the properties of hybrid nanofluids. Among the mixtures, Al_2_O_3_–Cu/EG–W was identified as the most effective and economic hybrid nanofluid for laminar flow, outperforming mono nanofluids at a mixing ratio of 20/80. The thermal conductivity enhancement at 20 °C was 0.482 W mK^−1^, but at a temperature of 60 °C, it was 0.542 W mK^−1^, indicating that as the temperature increases, the thermal conductivity also increases. However, the thermal conductivity is reducing at a mixing ratio of 50/50, indicating that the nanoparticle mixture ratio is important to enhance the thermal conductivity of hybrid nanofluids. Moreover, the study highlighted that the nanoparticle mixture ratio is important for determining the alignment of nanoparticles, leading to better heat transfer characteristics due to the synergistic effect, which means that there is no chemical reaction among the nanoparticles. The enhancement in thermal conductivity is based on the alignment and clustering of the nanoparticles. The study concluded that as the diameter of clusters and the viscosity of the base fluid increase, particularly in hybrid nanofluids, the heat transfers as the Brownian motion decreases, leading to a reduction in thermal conductivity. According to Ma *et al.*,^51^ the nanolayered structures form around nanoparticles in the liquid due to the ordered arrangement of liquid molecules influenced by van der Waals forces. These forces cause molecules to cluster into larger nanolayered structures with increased particle density and reduced thermal resistance. These structures act as thermal bridges, linking liquid molecules to nanoparticles and creating an intermediate state similar to a solid–liquid interface. The liquid molecules within these thermal bridges have properties that are intermediate between those of the nanoparticles and the liquid, thus enhancing the thermal conductivity of the nanofluids. Initially, van der Waals forces positively affect the thermal conductivity by facilitating the formation of clusters that enhance heat transfer. However, over time, the growing mass of these clusters may reduce thermal conductivity due to sedimentation. To counter this effect, physical agitation can be employed to prevent sedimentation and maintain the improved thermal conductivity of the nanofluids. The study indicates that dispersing nanoparticles of varying sizes into base fluids promotes the ordered arrangement of liquid molecules around the nanoparticles, leading to a more compact solid–liquid interface and significant improvements in the system's thermal performance.

Besides that, Rehman *et al.*^41^ have emphasised the importance of the surfactant in maintaining the stability and good thermal conductivity of the hybrid nanofluid. In this study, the effects of 8 different types of surfactants such as CTAB, SDS, PEG, oleyl amine (OLAm), poly (vinyl alcohol) (PVP), gum arabic (AG), PVA, and oleic acid (OA) on the stability and thermophysical properties of Al_2_O_3_/TiO_2_ at a mixing ratio of 20/80 with 0.02 vol% in water/EG (60 : 40) were analysed. Based on the result, PVP was selected as the best surfactant based on the good stability (60 days) and moderate viscosity and also due to the thermal conductivity enhancement of 3.6% at 80 °C and 8.5% viscosity enhancement. Based on the sedimentation method, the hybrid nanofluid with the CTAB, SDS, PEG and OA form sedimentation and agglomeration clearly, whereas the hybrid nanofluid with the PVP, AG, PVA and OLA are stable for more than 60 days without any sedimentation and agglomeration,^41^ whereas based on the UV-vis spectroscopy, PVP exhibited the highest absorbance peak followed by the Ag, OLA and hybrid nanofluid without surfactants at day 1, and even after 60 days, PVP show the highest adsorption peak compared to AG, PVA, OLAM and also hybrid nanofluids without the surfactant.^41^ It indicates that the PVP, AG and the hybrid nanofluid without the surfactant shows the stable hybrid nanofluid. Based on the stability, the trend after 60 days is PVP > without surfactant hybrid nanofluid > PVA > AG > OLAm > SDS > CTAB > PEG > and OA.^41^ It is obvious that PVP exhibited excellent stability when compared with other surfactants due to its non-ionic nature, providing electrical neutrality that makes it hydrophilic, longer alkyl chain, which aids the steric stability by generating strong repulsive forces between the nanoparticles and the base fluid, and higher molecular weight that allowed hybrid nanofluid to be stable at high temperatures.^41^ The least stability hybrid nanofluid is with the SDS surfactant, which causes the formation of bubbles and leads to a decrease in the suspension of nanoparticles. However, Rehamn *et al.*^41^ have come to a conclusion that the addition of surfactants in certain cases increases the stability but decreases the thermal conductivity. For example, the addition of OA and PEG causes high enhancement in thermal conductivity (8.3%), but it has poor stability which can sustain less than 10 days. However, PVA and PVP show good stability up to 60 days, but does not contribute for enhancement in thermal conductivity.

Not only that, Urmi *et al.*,^117^ also study the effect of different type of surfactant (PVP, SDBS and SDS) in the same type of hybrid nanofluid which is Al_2_O_3_/TiO_2_ based hybrid nanofluid in water/EG (60 : 40), but at different mixing ratio and volume fraction. In this study the mixing ratio of Al_2_O_3_ : TiO_2_ is 50 : 50 at 0.1 vol%. Without the surfactant, the zeta potential value of the hybrid nanofluid was 28 mV, but after the addition of surfactants such as PVP, it increased to 39.8 mV and the stability was maintained for 8 days. After 1 week, sedimentation started to appear. The highest stability is shown by the hybrid nanofluid with the addition of PVP compared to SDBS and SDS. However, the PVP-infused hybrid nanofluid results in a significant enhancement in thermal conductivity compared to the base fluid, with the highest enhancement (17.05%) observed at 70 °C. They also agreed that even though the addition of surfactant increases the stability, but at the same time it reduces the thermal stability.^117^ In a nutshell, Al_2_O_3_/TiO_2_ hybrid nanofluids produced in this work show great potential for improving heat transfer efficiency in various industrial applications, leading to more environmentally friendly and effective cooling solutions in the future. As a result, Urmi *et al.*,^118^ also further their study on the effect of different mixing ratio of TiO_2_ : Al_2_O_3_ (20 : 80, 40 : 60, 50 : 50, 60 : 40, 80 : 20) in water/EG (60 : 40), at constant volume fraction which is 0.1% volume concentration. They revealed that without the use of a surfactant, a very stable hybrid nanofluid is produced at Al_2_O_3_/TiO_2_ (20 : 80) for 21 days. The hybrid nanofluid's zeta potential is 34 mV, and its thermal conductivity is increased by 40.86%. Therefore, here the stability improvement is achieved by magnetic stirring and sonication to break down the agglomeration of particles and to archive a homogenous solution.^118^ Moreover, it has been discovered that the hybrid nanofluid with a high ratio of TiO_2_ achieved a larger value of absorbance based on UV-vis spectral analysis than a larger ratio of Al_2_O_3._ This is due to the van der Waals force among Al_2_O_3_. Based on this study, the ultrasonic vibration method was employed in this study without any surfactant or pH stabiliser, which is the main reason for the stability improvement.

Moreover, Wanatasanappan *et al.*^119^ investigated the effects of different mixture ratios of 20 : 80, 40 : 60, 50 : 50, 60 : 40 and 80 : 20 of Al_2_O_3_/Fe_2_O_3_ EG/water-based hybrid nanofluids on the viscosity and rheological properties. According to the findings, an Al_2_O_3_/Fe_2_O_3_-based hybrid nanofluid at a ratio of 40/60 showed the highest viscosity value at all temperatures, while the mixture ratio of 60/40 recorded the lowest viscosity value, and the hybrid nanofluid shows Newtonian fluid characteristics. As the temperature increases, the maximum viscosity reduction was 87.2%. In addition, the nanocomposite with a mixture ratio of 50/50 shows the highest zeta potential value and smaller particle size distribution with a greater pH value than the base fluid. Overall, it can be concluded that the effect of the nanoparticle composition ratio is less effective than the effect of temperature toward the viscosity.^119^ The Al_2_O_3_/Fe_2_O_3_ (40 : 60) ratio causes the highest dynamic viscosity enhancement of approximately 4.94%, whereas the temperature increase causes an impact of approximately 57.16%. Hence, it is clear that the base fluid's nanoparticle compositions contribute to the reduction in viscosity, which may be suppressed by raising the temperature of the nanofluid up to 87.1%. In addition, the base fluid's Newtonian fluid behaviour is unaffected by variations in the nanocomposite's mixing ratio. All hybrid nanofluid ratios behaved as Newtonian fluid for all temperatures from 0 to 100 °C and a shear rate between 5 and 4300 s^−1^. Therefore, it can be concluded that the Al_2_O_3_/Fe_2_O_3_ hybrid nanofluid with a ratio of 50/50 has a high potential to be used in the convective heat transfer application.

Besides, Jin *et al.*^46^ demonstrated that the incorporation of metal nanoparticles such as Cu or Ag into CNT composites create hybrid nanofluids results in higher thermal conductivity, improved stability, and lower viscosity compared to the functionalized CNT nanofluids. Specifically, Ag/CNT nanofluids exhibited superior thermal conductivity to Cu/CNT nanofluids, probably due to Ag's higher thermal conductivity. At 65 °C and a mass fraction of 0.05 wt%, Cu/CNT and Ag/CNT water-based hybrid nanofluids achieved maximum thermal conductivities of 0.8712 W m^−1^ K^−1^ and 1.0231 W m^−1^ K^−1^, respectively. The study found that the viscosity decreased with the increase in temperature but increased with higher mass fractions. This behaviour can be attributed to the reduction in intermolecular interactions between nanoparticles and the fluid at higher temperatures, which increases kinetic energy and lowers viscosity. Conversely, higher nanoparticle concentrations in the base fluid lead to increased viscosity due to enhanced internal viscous shear stresses. Additionally, at lower temperatures, increased Brownian motion may contribute to higher viscosity. Table 7 shows the summary of the different combinations of water/EG-based hybrid nanofluids.

**Table 7:** Type of hybrid W/EG-based nanofluids

NPs	W : EG	Vol%/ratio	Stability improvement	Stability/thermal conductivity (*T*_c_)	Ref.
Al_2_O_3_ : CuO	60 : 40	*φ*: 1.0% (20 : 80)	Mixture ratio/physical vibrations (magnetic stirrer for 15 min and ultra-sonication for 1 h)	*T*_c_: 12.64%	51
Al_2_O_3_ : TiO_2_	60 : 40	*φ*: 0.02% (20 : 80)	PVP	*T*_c_: 3.6% at 80 °C	41
				Stability: 60 days	
Al_2_O_3_ : TiO_2_	60 : 40	*φ*: 0.1% (50 : 50)	PVP	*T*_c_: 17.05% higher	117
				Stability: 8 days	
Al_2_O_3_ : TiO_2_	60 : 40	*φ*: 0.1% (20 : 80)	Ultrasonication method	*T*_c_: 40.86%	118
				Stability: 21 days	
Al_2_O_3_ : Fe_2_O_3_	60 : 40	(50 : 50)	PVP	—	119
Cu : CNT		*φ*: 0.05%	Functionalized CNT	*T*_c_: 8.82% and 11.94% (25 °C)	46
				*T*_c_: 52.37% and 40.02% (65 °C)	
Ag : CNT				Stability: no sedimentation up to 6 months	

### 7.3 Bio-based water or water/EG hybrid nanofluids (green hybrid nanofluids)

While recent research has explored hybrid nanofluids combining metal, metal oxide, and carbon nanoparticles, there are surprisingly very limited studies focusing on the use of plant-based nanomaterials in the synthesis of water-based hybrid nanofluids for various applications. This indicates that plant-based nanomaterials would be next novelty water-based hybrid nanofluids. Over the past decade, Industry 4.0 has integrated several renewable energy sectors through the development of virtual power plants and microgrids, enhancing the sustainability and cost-effectiveness of renewable power generation. Technologies applied in hydropower, wind, solar, geothermal, and biomass have all benefited from these advancements, boosting the production efficiency. The Sustainable Development Goals (SDGs) have greatly enhanced the global concept of “sustainability”, which is now relevant to nearly all areas of everyday life. Sustainable manufacturing and sustainable energy are essential elements of the 17 SDGs, specifically represented by SDG 12 and SDG 7, which closely linked to the nanotechnology revolution.

**Fig. 26:**
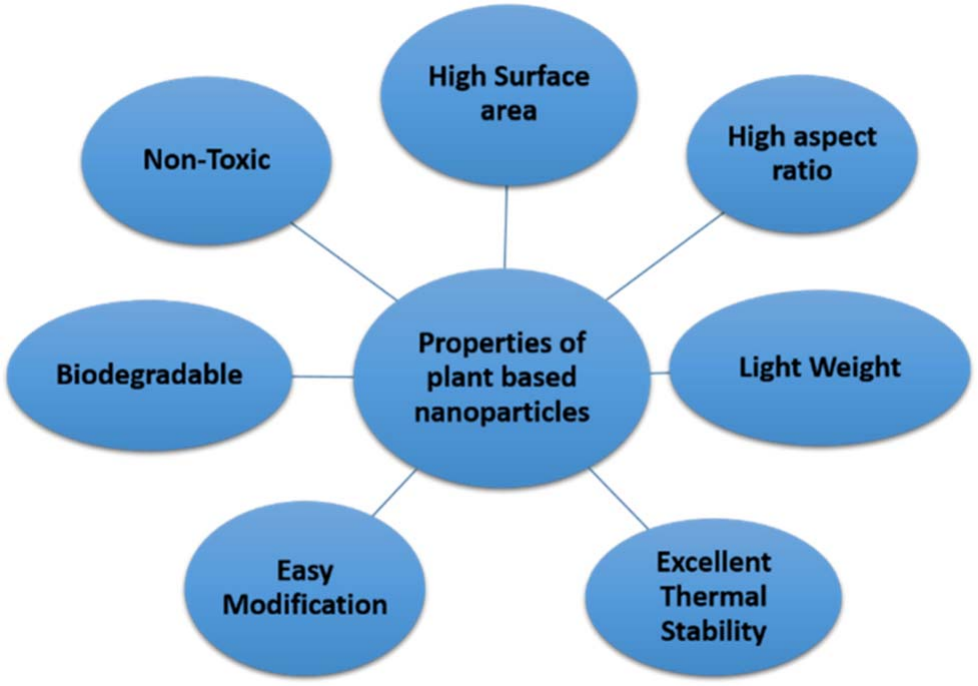
Properties of plant-based nanomaterials that make them suitable for application in hybrid nanofluids.

The rising demand for environmentally friendly, biodegradable, and biocompatible materials has led to the exploration of plant-based nanomaterials such as nanocellulose. Nanocellulose, with its high hydrophilicity, is emerging as a viable sustainable material for various applications.^120^ Harun *et al.*^121^ have reviewed the preparation of nanocellulose for new green working fluids in heat transfer applications including car radiators, cutting tool coolants, and pool boiling. This is because the preparation of nanofluid is very crucial because improper preparation will lead to the deterioration of nanofluid and decrease the thermal performance as a heat transfer working fluid. In addition, low stability causes nanoparticles to form clusters and sediments due to its strong van der Waals interaction. Therefore, according to the literature review, ultrasonication for 2 to 3 hours is the optimum duration, as it will emits supersonic waves travel longitudinally within the liquid medium and caused the alternate positive and negative pressure waves in the liquid. Thus, this will increase the stability of nanofluids. Moreover, most of the literature used a low concentration of nanocellulose, due to the stability issue which could cause clogging during operation. Moreover, the two-step method is widely used in most of the studies reported in the literature as it is much simpler and more economic, compared to the one-step method. Despite its lack of inherent conductivity, nanocellulose's unique properties make it a strong candidate for hybrid nanofluid applications. Notably, its ability to undergo surface modification enhances its compatibility with other nanoparticles, helping achieve the desired properties for hybrid nanofluids. Additional properties that contribute to the suitability of nanocellulose for hybrid nanofluids are illustrated in Fig. 26.

Plant-based cellulose nanoparticles known as nanoscale have high surface area and light weight, which have the ability to increase the reactivity and interaction with the other materials.^122^ Harun *et al.*^49^ have also conducted a study on the effect of surfactants in nanocellulose water-based nanofluids with and without the addition of surfactant named Triton X-100, which is more suitable for non-metallic materials like plant-based nanoparticles at different volume fractions of nanocellulose in water. The result shows that the highest thermal conductivity was 1.224 W m^−1^ K^−1^ for 0.5 vol% at 40 °C. Moreover, the nanocellulose water-based nanofluid with surfactants was stable even after 2 weeks, due to the steric stabilization of Triton X-100, where the polymeric chains get absorbed on the nanocellulose surface, free movement of the nanoparticle in the base-fluid is restricted and this acts as a steric diffusion barrier to prevent nanoparticle agglomeration.^37^ Ramachandran *et al.*^123^ have synthesised a nanofluid using nanocellulose with a weight concentration of 7.4% in the EG/water (40/60%) mixture. The nanofluid has maximum thermal conductivity enhancement of 9.05% or 0.479 W m^−1^ K^−1^ at 1.3% volume concentration when it is compared to the base fluid at 30 °C. After magnetic stirring for 1 hour and treatment with ultrasonic waves for 2 h, the suspension of nanocellulose in the base fluid was observed for dispersion stability and it appeared to be stable for more than a month. This indicates that the addition of plant-based nanoparticles (nanocellulose) only in water or in EG/water can increase the thermal conductivity and stability of nanofluids. Besides that, Naiman *et al.*^124^ have studied the effect of incorporation of nanocellulose with Al_2_O_3_ in a car radiator using EG/water at different concentrations of 0.1%, 0.5% and 0.9%. The result revealed that the addition of nanocellulose provides better heat transfer efficiency than the use of distilled water as a radiator coolant. It was found that 0.5% of volume concentration is the better rate of heat transfer than that of distilled water.

**Fig. 27:**
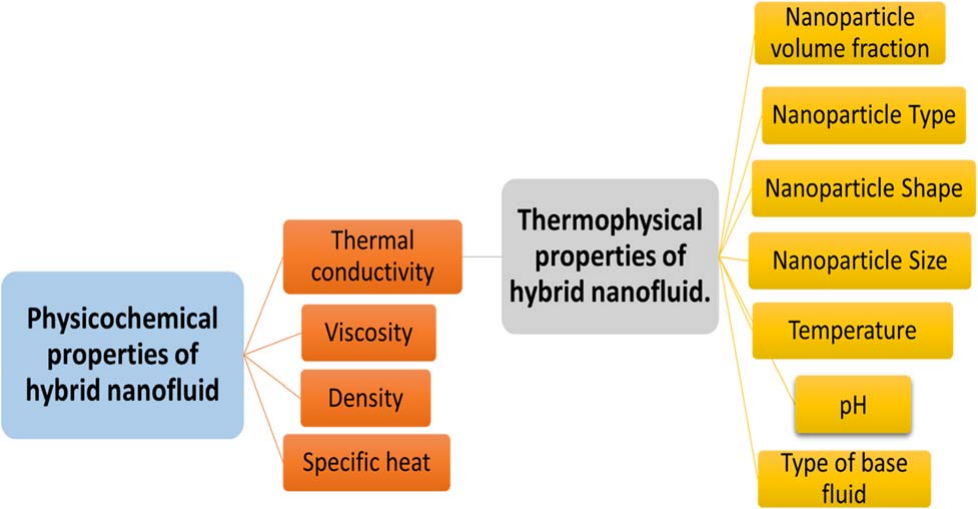
Physicochemical and thermophysical properties play a crucial role in determining the characteristics and performance of hybrid nanofluids.

Furthermore, Yaw *et al.*^125^ produced a hybrid nanofluid composed of graphene nanoparticles (GNPs) and cellulose nanocrystalline (CNC) in 40/60 ratio of distilled water and ethylene glycol. They proposed this type of hybrid nanofluid improve the efficiency of heat transfer of a vehicle radiator with only a small addition of 0.01% GNP/CNC. Sandhya *et al.*^126^ produced a similar type of hybrid nanofluid using CNC and GNP in a mixing ratio of 50/50 at different volume fractions ranging from 0.01% to 0.2% for heat transfer applications. Based on their study, 0.1% was identified as the optimal volume fraction, providing excellent colloidal stability in an EG/water base fluid. The stability was maintained for 30 days without any sedimentation. This hybrid nanofluid was synthesized without the addition of surfactants, as surfactants can create bubbles and contaminate heat transfer channels, negatively affecting the overall performance of the heat transfer device. To enhance the stability, a magnetic stirrer and 5 hours of probe sonication were used. The chemical modification of nanocellulose is another way to increase the stability. Techniques such as oxidation, esterification, etherification, silane grafting, acetylation, carboxymethylation, and alkylation are commonly used, but they do not require coupling agents or surfactants. Functionalization serves two main purposes: one is to induce anionic charges on the surface of nanocellulose, achieved through phosphorylation, carboxymethylation, oxidation, and sulfonation; the other is to create a hydrophobic surface, accomplished through acetylation, silylation, urethanization, and amidation.^127^ Last but not least, it is clear that thermal conductivity is influenced by thermophysical properties such as nanoparticle volume fraction, nanoparticle type, shape, size, temperature, pH value and type of base fluid, as summarized in Fig. 27.

#### 7.3.1 Future direction

Future research on hybrid nanofluids must prioritize the utilization of plant-based nanomaterials such as nanocellulose to develop sustainable and cost-effective hybrid nanofluids. Currently, research in this area is highly limited. The use of nanocellulose in hybrid nanofluids should be expanded by leveraging available biomass wastes such as oil palm, rice fields, and kenaf from industries. Additionally, little attention has been paid to the aging and reusability of nanofluids—both mono and hybrid—in future applications. When considering the real-world implementation of nanofluids, two key challenges emerge: erosion and sedimentation. These issues must be thoroughly investigated and addressed before nanofluids can be widely adopted for commercial use. Further studies should focus on determining the upper limits of volume fraction and optimizing the nanoparticle ratio in composite powders to maximize the performance.

Moreover, the relationship between stability and thermal conductivity remains an underexplored area. Since these properties are interrelated, achieving good stability in hybrid nanofluids is crucial for maintaining high thermal conductivity. Poor stability, often due to nanoparticle coagulation, negatively impacts thermal performance, limiting the heat transfer potential of hybrid nanofluids. Therefore, stability assessment must be an integral part of future research, as inadequate stability can alter the thermophysical properties of hybrid nanofluids, leading to poor heat transfer efficiency. Several methods including centrifugation, sedimentation, zeta potential analysis, electron microscopy, UV-vis spectroscopy, and light scattering techniques have been developed for stability analysis. Ensuring long-term nanoparticle suspension remains a significant challenge in nanofluid research. Two primary concerns must be addressed: (1) preparing homogeneous and long-term stable nanofluids and (2) maintaining the initial equilibrium before measuring thermal conductivity. Poor dispersion stability is a major factor contributing to a decline in thermal performance in heat transfer applications. Additionally, an increase in both volume fraction and temperature generally enhances thermal conductivity, as confirmed by most studies. However, limited research has demonstrated the possibility of improving thermal conductivity at lower temperatures. It has been observed that while hybrid nanofluid properties tend to improve with the increase in temperature and volume fraction, some nanofluids exhibit upper limits beyond which performance deteriorates at higher concentrations. Generally, thermal conductivity enhancement increases monotonically with particle loading, but the effect of temperature varies depending on the type of nanofluid. It is important to ensure that the base fluid and nanofluid temperatures are the same when comparing thermal conductivity values, as a higher base fluid conductivity can reduce the enhancement ratio. To improve the hybrid nanofluid stability, researchers commonly employ surface modification techniques, magnetic stirring, ultrasonication (both direct and indirect), and, in some cases, surfactants. Meanwhile, optimizing factors such as nanoparticle type, ratio, volume fraction, pH, and temperature is crucial for enhancing the thermal stability. The literature suggests that inconsistencies in the thermophysical properties of nanofluids may arise from variations in surfactant type and concentration. However, further studies are needed to confirm the impact of surfactants on hybrid nanofluid properties. In conclusion, achieving long-term stability remains a major challenge hindering the commercial development of nanofluids. Unstable nanoparticle agglomeration is a key factor contributing to inconsistencies in reported thermal conductivity and viscosity values. Therefore, future research must focus on identifying the optimal nanoparticle concentration that minimizes viscosity while maximizing thermal conductivity.

Last but not least, future studies should address the expected changes in non-water-based nanocomposites used for heat transfer applications, including those in the biomedical field. Heat transfer plays a crucial role in therapies such as hyperthermia for cancer treatment, cryotherapy, and laser-tissue interactions. In some cases, non-water-based nanocomposites, such as CNTs or graphene, are commonly used to enhance thermal conductivity and mechanical properties. However, dispersion challenges arise when these nanocomposites are introduced into water. This issue can be mitigated through the addition of surfactants or dispersion agents, surface modification, or high-shear mixing. Furthermore, beyond studies on the stability and thermal conductivity of hybrid nanofluids, further research on rheological properties—such as viscosity and polymer relaxation time—is essential to better assess heat transfer performance.

## 8 Conclusion

In summary, this review highlighted that hybrid nanoparticles significantly enhance the physicochemical properties of nanofluids compared to single nanoparticles. Achieving a stable nanoparticle dispersion is essential for optimizing the thermal conductivity of hybrid nanofluids, as stability and thermal conductivity are closely interrelated. Long-term stability prevents particle aggregation and sedimentation, which can otherwise degrade thermal properties and heat transfer efficiency. Researchers have explored various strategies to improve both stability and thermal conductivity, including surfactant addition, optimization of sonication time, enhancement of repulsive forces, and surface modification. Each method has its advantages and limitations. For instance, while surfactants generally improve stability, they may also reduce thermal conductivity. Thus, selecting the appropriate surfactant and concentration is crucial, as some additives can degrade thermophysical properties, cause foaming at high temperatures, and reduce performance.

Surface modification appears to be a particularly promising technique for enhancing the long-term stability of hybrid nanofluids, offering more benefits than drawbacks. External forces such as sonication and magnetic stirring also help mitigate agglomeration; however, optimizing the sonication time is necessary to prevent any adverse effects on nanofluid properties. Proper sonication can improve stability and, consequently, thermal conductivity. Additionally, the choice of nanoparticle types, their ratios, and volume fractions plays a critical role in the synthesis of hybrid nanofluids. Each nanoparticle type has distinct properties that influence heat transfer performance when incorporated into water-based fluids. Thermal conductivity is affected by temperature and nanoparticle volume fraction, and optimizing these parameters can enhance the overall efficiency. As the temperature increases, Brownian motion intensifies, promoting better particle dispersion and improving thermal conductivity. However, viscosity is directly influenced by temperature and concentration, requiring careful control to maintain optimal performance. With the growing focus on sustainability, expanding research on plant-based nanoparticles in hybrid nanofluids is highly recommended, as studies in this area remain limited. Ultimately, selecting the most suitable hybrid nanofluid for various applications requires balancing cost-effectiveness, stability, and efficient heat transfer performance.

## Data availability

No primary research results, software or code have been included and no new data were generated or analysed as part of this review.

## Conflicts of interest

The authors state no conflict of interest.
